# β‐Mangostin Attenuates TET2‐Mediated DNA Demethylation of Prkcg in the Prevention of Intervertebral Disc Degeneration

**DOI:** 10.1002/advs.202505077

**Published:** 2025-06-25

**Authors:** Xiangzhen Kong, Hanwen Gu, Yuanqiang Zhang, Qunbo Meng, Qi Li, Kangle Song, Yanlin Li, Kaiwen Liu, Zhenchuan Liu, Rui Hu, Haoxi Zhai, Tian Li, Zemin Ling, Zhijian Wei, Fuxin Wei, Lei Cheng

**Affiliations:** ^1^ Department of Orthopaedic Surgery Qilu Hospital Cheeloo College of Medicine Shandong University Jinan Shandong 250012 P. R. China; ^2^ The First Clinical College of Shandong University Jinan Shandong 250012 P. R. China; ^3^ Tianjin Key Laboratory of Acute Abdomen Disease‐Associated Organ Injury and ITCWM Repair Institute of Integrative Medicine of Acute Abdominal Diseases Tianjin Nankai Hospital Tianjin Medical University 8 Changjiang Avenue Tianjin 300100 P. R. China; ^4^ Shenzhen Key Laboratory of Bone Tissue Repair and Translational Research Department of Orthopaedic Surgery The Seventh Affiliated Hospital of Sun Yat‐sen University Shenzhen 518107 P. R. China; ^5^ Department of Orthopaedics Qilu Hospital of Shandong University Shandong University Centre for Orthopaedics Advanced Medical Research Institute Shandong University No. 107 Wenhua West Road, Lixia District Jinan 250012 P. R. China; ^6^ Department of Orthopaedics The Second Hospital of Shandong University No. 247 Beiyuan Street, Tianqiao District Jinan 250033 P. R. China; ^7^ Department of Orthopedics Tianjin Medical University General Hospital International Science and Technology Cooperation Base of Spinal Cord Injury Tianjin Key Laboratory of Spine and Spinal Cord Injury No. 154 Anshan Road, Heping District Tianjin 300052 P. R. China

**Keywords:** β‐mangostin, demethylation, intervertebral disc degeneration, Prkcg, TET2

## Abstract

Intervertebral disc degeneration (IDD) induced lower back pain is a main cause of disability, resulting in a substantial workforce loss worldwide and placing a substantial burden on the global economy and healthcare systems. However, no effective disease‐modifying therapies presently exist for IDD or its related pathologies. Single‐cell sequencing analyses reveal progressive M1 macrophage polarization in NP cells correlating with IDD severity, underscoring the therapeutic imperative for dual‐targeting agents addressing both inflammatory dysregulation and matrix homeostasis. β‐mangostin (β_Man) is screened to be proven to possess potential therapeutic effects in alleviating IDD. β_Man possesses anti‐inflammatory capabilities, which include remodeling the homeostasis of the extracellular matrix, regulating macrophage polarization, and inhibiting apoptosis in the nucleus pulposus. TET2‐Prkcg exerts significant regulatory functions downstream of β_Man. Mechanically, β_Man mediated reduction of TET2 maintains the DNA methylation of Prkcg rather than hydroxymethylation, which promotes mitophagy and alleviates the inflammatory microenvironment. β_Man represents a promising novel therapeutic strategy for IDD treatment. The TET2‐Prkcg axis emerges as a novel therapeutic target for IDD treatment.

## Graphical abstract

β‐Mangostin alleviates intervertebral disc degeneration through dual modulation of macrophage polarization and the TET2‐Prkcg axis in nucleus pulposus cells.

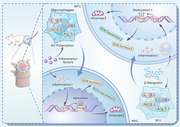

## 1 Introduction

Lower back pain (LBP) is a leading cause of disability.^[^ ^1^ ^]^ It has resulted in heavy economic and health burdens, with two estimates of indirect costs in the United States being $1.85 billion and $2.82 billion in 1996.^[^ ^2^ ^]^ The global number of years lived with a disability caused by LBP increased by 54% from 1990 to 2015, the primary cause of which is intervertebral disc degeneration (IDD).^[^ ^3^ ^]^ Additionally, natural aging contributes to IDD, and senescent cells secrete endogenous inflammatory substances such as IL‐1β, IL‐6, and IL‐8 in the intervertebral disc (IVD).^[^ ^4^ ^]^ Immune cell infiltration by macrophages and neutrophils forms an inflammatory microenvironment in the disc.^[^ ^5^ ^]^ This results in extracellular matrix (ECM) dysfunction, a reduction in the number of functional cells, decreased proteoglycan content, dehydration, and calcification.^[^ ^6^ ^]^ These mechanisms highlight the criticality of the homeostatic regulation of the IVD microenvironment as a crucial step in IDD treatment. However, no effective disease‐modifying therapies exist at present for IDD or its associated pathologies. Natural products have substantial potential as novel agents for addressing these therapeutic challenges.

Drug‐screening approaches can leverage anti‐inflammatory properties to regulate the inflammatory microenvironment and address ECM metabolism. Targeting the key genes related to IDD and modulating their expression are other effective therapeutic interventions. β‐Mangostin (β_Man), a xanthone derivative extracted from mangosteen fruit,^[^ ^7^ ^]^ demonstrates various biological functions. β_Man protects against apoptosis via the MEK/ERK and p53 signaling pathways in the retina.^[^ ^8^ ^]^ Furthermore, β_Man mitigates oxidative stress by modulating the PI3K/AKT/mTOR pathway.^[^ ^9^ ^]^ Notably, it enhances selective autophagy and facilitates melanosome degradation.^[^ ^10^ ^]^ β_Man has shown anticancer properties, including inhibiting glioma progression via STIN pathway activation,^[^ ^11^ ^]^ and reducing cervical cancer metastasis by suppressing the JNK2/AP‐1/Snail signaling cascade.^[^ ^12^ ^]^ β_Man is considered to demonstrate great therapeutic potential in IDD treatment.

Protein kinase C is a serine/threonine‐protein kinase, and protein kinase C gamma type (Prkcg) is one of its isoforms.^[^ ^13^ ^]^ which regulates intracellular signal transduction in different cellular processes.^[^ ^14^ ^]^ Prkcg is highly expressed in the Purkinje fibers of the cerebellum,^[^ ^15^ ^]^ and its mutations result in cerebellar ataxia.^[^ ^16^ ^]^ Prkcg downregulation can effectively alleviate LPS‐induced sepsis,^[^ ^17^ ^]^ reduce inflammatory astrocyte proliferation, improve functional recovery following spinal cord injury,^[^ ^18^ ^]^ and inhibit mechanical pain caused by inflammation.^[^ ^19^ ^]^ Its ability to regulate signal transduction makes it a primary target for drugs such as Scutellarin and Metformin.^[^ ^20^ ^]^ However, Prkcg has not yet been explored in vitro, especially in bone and cartilage research. It has only been reported to enhance expression in arthritis.^[^ ^21^ ^]^

Herein, we demonstrated the crucial role of Prkcg in IDD. Additionally, the natural compound β_Man targets Prkcg as its downstream target and is revealed to inhibit IDD progression. β_Man attenuated the inflammation‐induced increase in TET2 expression, which could silence Prkcg by increasing DNA methylation, promoting IVD regeneration and repair. These results provide direction for IDD treatment.

## 2 Results

### 2.1 ECM Metabolic Disruption and Macrophage Infiltration in Degenerated Nucleus Pulposus

**Figure 1:**
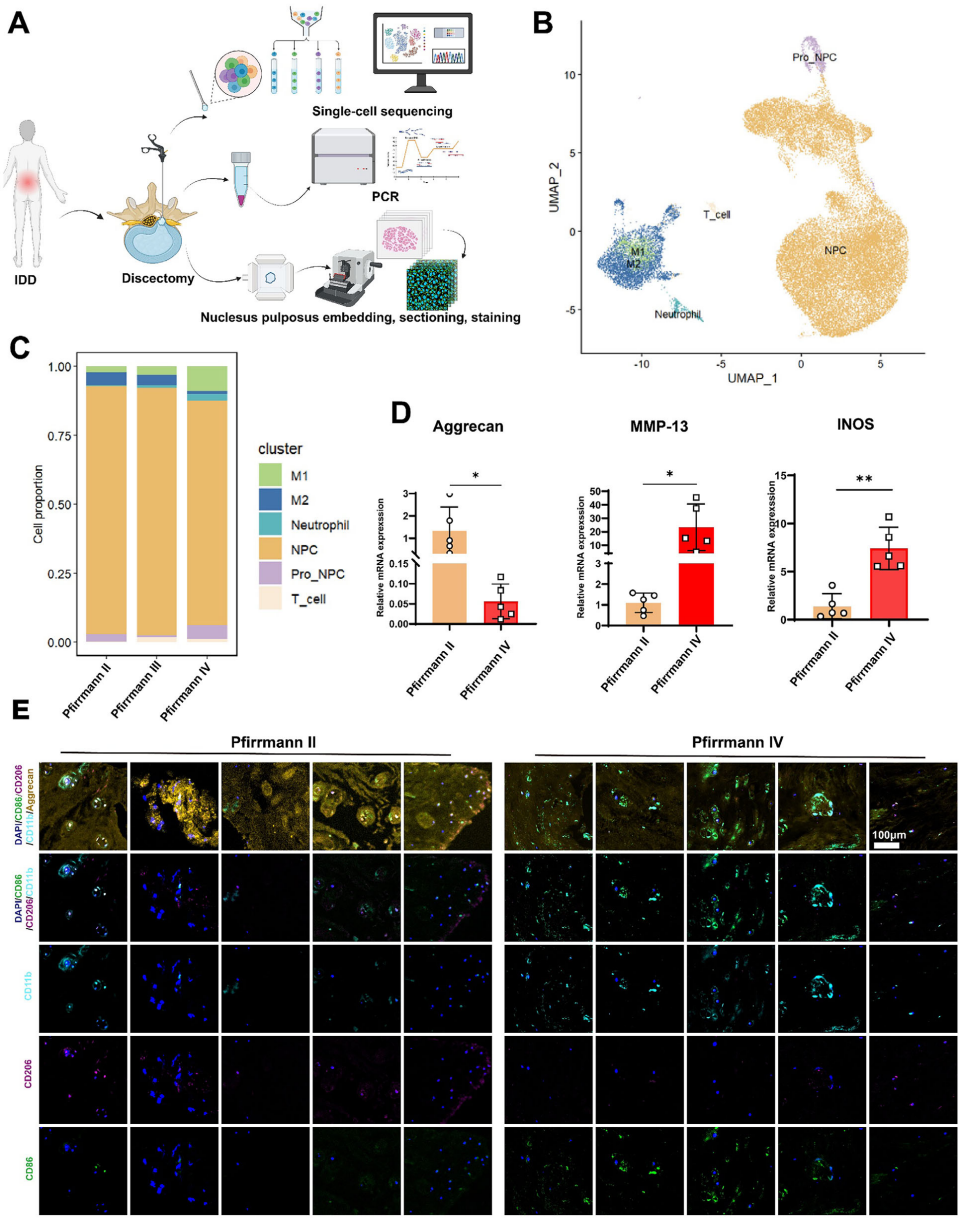
ECM metabolic disruption and macrophage infiltration in degenerated NP. A) Collection of NP sample and experimental workflow diagram (created using BioRender.com). B) UMAP map of the following 5 cell types: T cells, NPCs, Neutrophils, progenitor NP cells (Pro_NPC), M1 and M2 macrophages. C) The proportion of various cell types in different degenerated NP. D) PCR detection for ECM metabolic changes in NP (n = 5). E) Representative IF images of Aggrecan, CD11b, CD206, and CD86 (n = 5, scale bar: 100 µm). Data is presented as mean ± SD. ∗*p* < 0.05, ∗∗*p* < 0.01, ∗∗∗*p* < 0.001.

Macrophages have been shown to influence IDD progression.^[^ ^22^ ^]^ To better visualize the relationship between IDD and macrophage infiltration or polarization, we collected NP samples of human beings and classified them using the Pfirrmann grading system (Table S1, Supporting Information) based on preoperative radiology text (**Figure** **1**A). Single‐cell sequencing reveals varying degrees of macrophage infiltration in degenerated NP (Figure 1B). There is an observable trend of a declining proportion of M1‐type macrophages concomitant with an augmentation in M2‐type macrophages as the progression of degeneration severity (Figure 1C). Next, we utilized PCR to evaluate ECM metabolism in NP, and the results revealed that the sample with a higher degree of degeneration displayed significant metabolic dysregulation and elevated inflammatory expression (Figure 1D). To investigate further the distribution of macrophages, immunofluorescence (IF) was used revealing that only a few macrophages were labeled in grade II degenerated NP, with no obvious polarization tendency (Figure 1E). In contrast, NP in grade IV exhibited greater macrophage infiltration and a stronger tendency for M1 polarization.

### 2.2 β_Man is a Potential Drug Candidate for the Treatment of IDD

**Figure 2:**
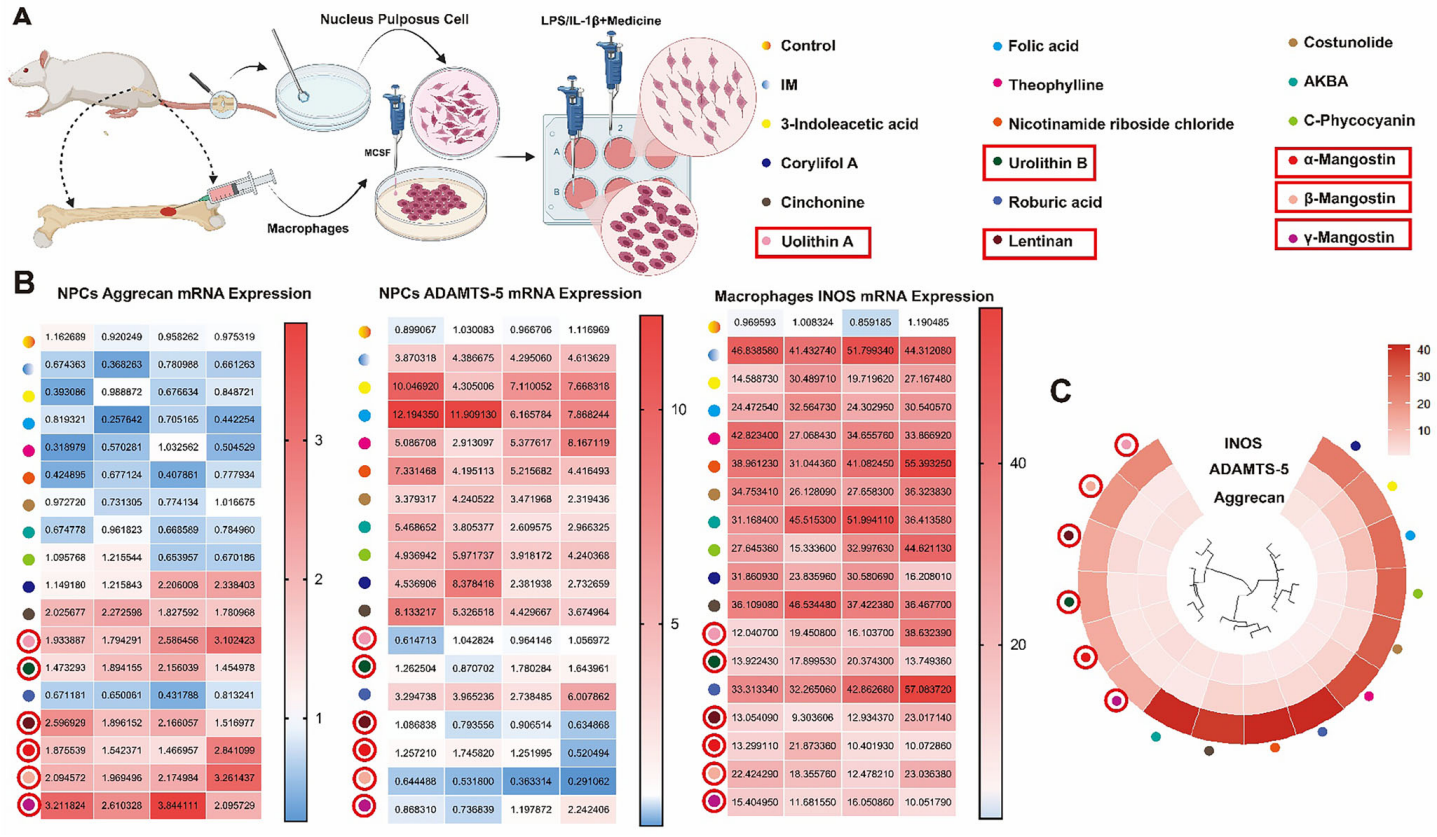
β_Man is a potential drug candidate for the treatment of IDD. A) Drug screening flowchart (created using BioRender.com). B) PCR analysis of mRNA expression for related genes (n = 4). C) Comparison of the effects of different drugs.

Modulating macrophage polarization and ECM metabolic disturbances is known to be crucial for improving IDD. We conducted a comprehensive drug screening to identify natural products capable of targeting two pathways (**Figure** **2**A). We selected plant extracts previously validated for their anti‐inflammatory activity in other fields. A total of 16 compounds were selected: 3‐indoleacetic acid, Folic acid, Theophylline, Corylifol A, Cinchonine, Costunolide, Urolithin A, Urolithin B, Nicotinamide riboside chloride, C‐phycocyanin, Roburic acid, Lentinan, α‐Mangostin, β‐Mangostin, γ‐Mangostin and AKBA. We utilized nucleus pulposus cells (NPCs) to model an inflammatory microenvironment in vitro using IL‐1β stimulation (IM group). Rat macrophages were extracted and used to induce M1 polarization by LPS. Then cells were exposed to the selected compounds following inflammation treatment. The effects were assessed by quantifying the mRNA expression levels of Aggrecan and ADAMTS‐5 in NPCs, and INOS in macrophages via PCR (Figure 2B). The results indicated that six natural compounds—Urolithin A, Urolithin B, Lentinan, α‐Mangostin, β‐Mangostin, and γ‐Mangostin—demonstrated promising therapeutic potential for further investigation (Figure 2C). We selected β_Man (Figure S1A, Supporting Information) for further investigation.

### 2.3 β_Man was Selected and Shown to Alleviate the Progression of IDD in Rats

**Figure 3:**
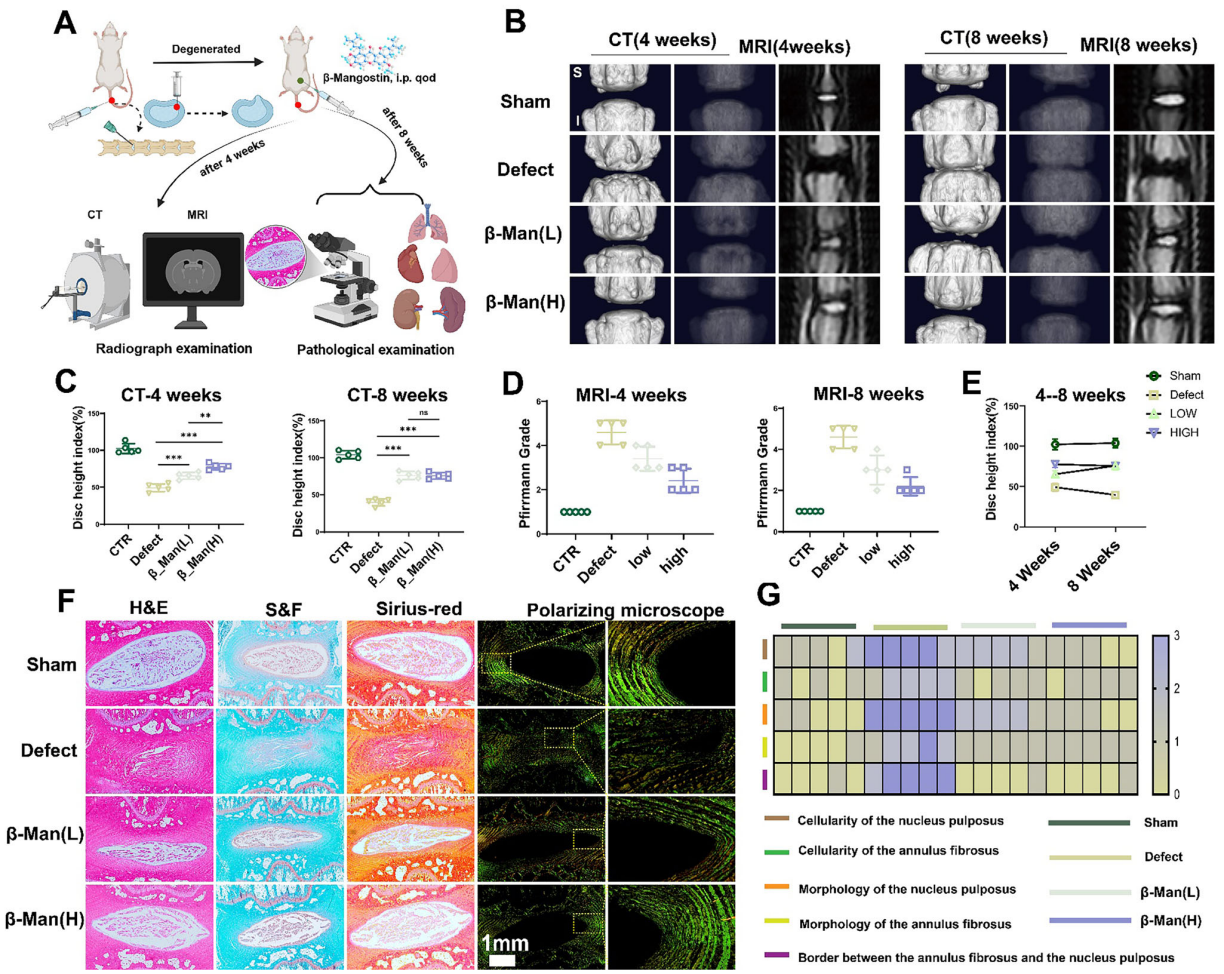
β_Man effectively restrains IDD progression in a rat tail needle puncture model. A) Schematic of the in vivo experimental workflow (created using BioRender.com). B) CT and MRI imaging of IVD in weeks 4 and 8 (S: superior, I: inferior). C) DHI calculations were performed using CT at 4 and 8 weeks. D) Pfirrmann grading was used to assess disc degeneration at 4 and 8 weeks. E) Changes in DHI at different times. F) Histological analysis of IVD (scale bar: 1 mm). G) Heatmap illustrating changes in histological scores across groups. Data is presented as mean ± SD, n = 5. ∗*p* < 0.05, ∗∗*p* < 0.01, ∗∗∗*p* < 0.001. ns, no significance.

We established a rat tail puncture model to verify further the therapeutic effect of β_Man on IDD in vivo. The rats were divided into four groups based on different treatments: the Sham group (only punctures the skin), the Defect group (normal saline therapy), the low‐dose treatment group (β_Man(L)), and the high‐dose treatment group (β_Man(H)). The modeling and treatment process is shown in **Figure** **3**A. Radiological examination assessments were performed in 4 and 8 weeks (Figure 3B). Pfirrmann grading and Disc Height Index (DHI) were used to evaluate disc degeneration levels and disc height (Figure S1E, Supporting Information). The results indicated that the Defect group had significant reductions in water content and disc height. In contrast, both treatment groups exhibited improvement, with the β_Man(H) slightly outperforming the β_Man(L) (Figure 3C,D). Interestingly, the β_Man(H) displayed a higher DHI at 4 weeks but had converged at 8 weeks (Figure 3E).

Pathological staining, including H&E, Safranin O/Fast Green, and Sirius Red staining combined with the polarized microscope, was used to evaluate the histology of IVD at 8 weeks.^[^ ^23^ ^]^ H&E staining of the heart, liver, spleen, lungs, and kidneys revealed no significant differences among the groups, demonstrating the biological safety of β_Man (Figure S1B, Supporting Information). The results showed that the Sham group exhibited a clear IVD structure, with the NP being round, and its internal structure neatly arranged. The AF was evenly distributed around the NP, with natural and distinct boundaries. Safranin O/Fast Green staining revealed abundant red staining of proteoglycans in the NP, while the AF and bone exhibited high levels of blue‐staining collagenous fiber. Sirius Red staining combined with the polarized microscope showed that the main collagen components in the AF were COL‐1 (yellow) and COL‐3 (green). COL‐2, the main component of the NP, had no color under the polarized microscope. In the Defect group, significant degeneration of the NP was observed. Most of the NP was replaced by fibrous structures, and the internal structure was disorganized. Both the β_Man(L) and β_Man(H) groups showed varying degrees of recovery, with the β_Man(H) group performing better than the β_Man(L) (Figure 3F,G). IHC analysis revealed reduced COL‐2α1 expression in the NP of the Defect group, accompanied by increased MMP‐13 and INOS expression. In contrast, both the β_Man(L) and β_Man(H) groups exhibited increased expression of COL‐2α1 and decreased expression of MMP‐13 and INOS (Figure S1C,D, Supporting Information). These findings indicate that β_Man can mitigate disc height loss and NP dehydration, reduce NP matrix degradation or fibrosis, and promote IVD regeneration and repair. Finally, we used CD11b to examine the infiltration of macrophages in the IVD (Figure S1C, Supporting Information). Interestingly, no significant positive results were observed in any group. This might be attributed to the short timeframe, which may have limited effective macrophage infiltration.

### 2.4 β_Man Mitigates Inflammation, Regulates ECM Metabolism, and Alleviates Apoptosis in NPCs and Annulus Fibrosus Cells

**Figure 4:**
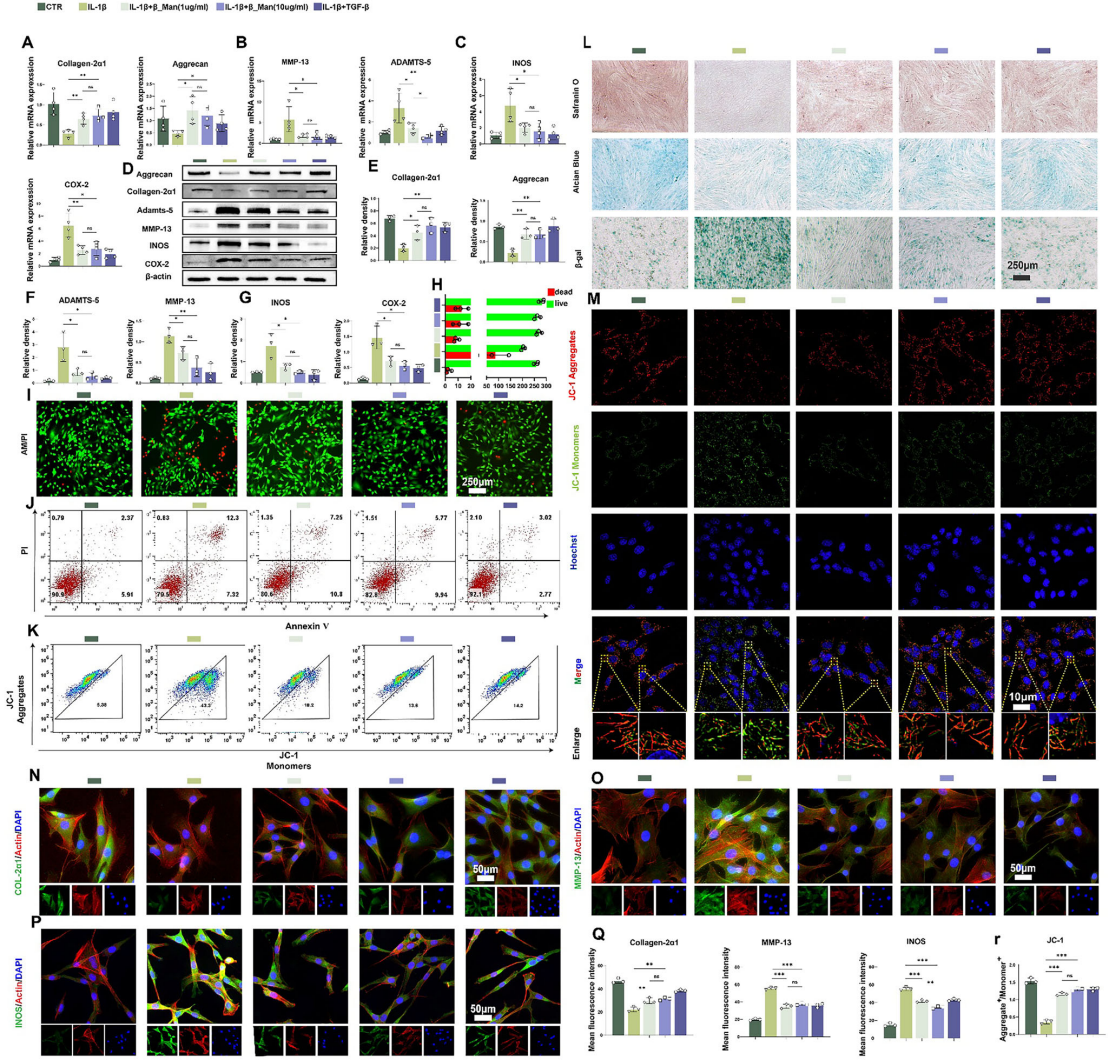
β_Man improves NPCs ECM metabolism, alleviates cellular senescence, and reduces apoptosis. A–C) PCR analysis of ECM metabolism and inflammation in NPCs (n = 4). D) Western blot shows the protein expression of the above genes (n = 3). E–G) Quantitative analysis of Western blot results. H) Quantitative analysis of live/dead staining results. I) Live/dead staining of NPCs (n = 3, scale bar: 250 µm). J) Flow cytometry of NPCs apoptosis under different treatments (n = 3). K) Flow cytometry of JC‐1 (n = 3). L) Safranin O, Alcian Blue, and β‐gal staining (n = 3, scale bar: 250 µm). M) JC‐1 staining of NPCs (n = 3, scale bar: 10 µm). N,O,P) IF staining of Collagen‐2α1, MMP‐13, INOS in NPCs (n = 3, scale bar: 50 µm). Q) Quantitative analysis of IF. R) Quantitative analysis of JC‐1 results. Data is presented as mean ± SD. ∗*p* < 0.05, ∗∗*p* < 0.01, ∗∗∗*p* < 0.001. ns, no significance.

We used IL‐1β to stimulate NPCs or AFCs, and establish an inflammatory degeneration model, to evaluate the protective effects of β_Man on NPCs (AFCs). TGF‐β, which is used as a positive control, is well‐known for its anti‐inflammatory properties and ability to ameliorate ECM metabolic disorders, exerting beneficial effects on both NPCs and AFCs.^[^ ^24^ ^]^ First, we performed a CCK‐8 assay to assess the toxicity of β_Man on AFCs and NPCs at different concentrations. The results showed no significant toxicity in cells cultured for 24 or 48 h at concentrations below 10 µg mL^−1^ (Figure S2B,C, Supporting Information). We selected high (10 µg mL^−1^) and low (1 µg mL^−1^) concentrations for the experimental treatments. PCR showed that β_Man improves the ECM metabolism disorder (**Figure** **4**A–C, Figure S3A, Supporting Information). The difference was that, for AFCs, we used Collagen‐1α1 instead of Collagen‐2α1 in the ECM synthesis gene expression analysis. The Western blot showed that the protein expression roughly agreed with the PCR results (Figure 4D–G, Figure S3B–E, Supporting Information). To better visualize the anti‐inflammatory effects of β_Man, we performed Safranin O staining, Alician blue, and β‐gal staining, and results indicated that β_Man effectively mitigated the IL‐1β‐induced reduction in collagen synthesis and aggravated cellular senescence in NPCs (AFCs) (Figure 4L, Figure S3F, Supporting Information). IF also showed that β_Man increased the expression of Collagen‐2α1 in NPCs and Collagen‐1α1 in AFCs while reducing the expression of MMP‐13 and INOS (Figure 4N–Q, Figure S3I–L, Supporting Information).

During IDD, macrophages are often recruited from the peripheral blood in IVD.^[^ ^25^ ^]^ Modulating macrophage polarization can improve the inflammatory microenvironment, forming a crucial basis for protecting IVD.^[^ ^26^ ^]^ We conducted targeted investigations into macrophage direction of polarization to investigate whether β_Man mediates an alternative macrophage‐dependent protective mechanism within the IVD system. CCK‐8 showed no significant toxicity in cells cultured for 24 or 48 h at concentrations below 10 µg mL^−1^ (Figure S2A, Supporting Information). We next used LPS‐induced M1 macrophages treated with β_Man and performed PCR to assess polarization. The results showed that β_Man effectively reduced the expression of INOS and IL‐1β while increasing the CD206 and Arg‐1 (Figure S4A, Supporting Information). However, macrophages did not exhibit significant polarization characteristics when β_Man was added alone. In the following step, IF (Figure S4B, Supporting Information) and Flow Cytometry (FCM) (Figure S4C,D, Supporting Information) were used and showed that β_Man effectively reduced CD86^+^ cells with LPS‐induced. Meanwhile, the ratio of CD86^+^/CD206^+^ cells was significantly reduced (Figure S4E, Supporting Information). However, no significant changes were observed in macrophages without LPS induction. These findings naturally indicated that β_Man effectively attenuates M1 polarization in LPS‐induced macrophages but does not directly promote M2 polarization.

We demonstrated that β_Man exhibits the potential to alleviate IDD via macrophage polarization regulation. Building on these mechanistic insights, we established a co‐culture system of macrophages with NPCs and AFCs to delineate macrophage‐NPC (AFC) crosstalk under β_Man treatment. Macrophages were seeded in transwell inserts and polarized to the M1 polarization via LPS stimulation (Figure S5A, Supporting Information). Following 72‐h β_Man treatment, conditioned medium was refreshed before establishing compartmentalized co‐culture with pre‐seeded NPCs or AFCs in 6‐well plates, enabling systematic investigation of macrophage‐mediated paracrine effects on cell viability and matrix homeostasis. TGF‐β can regulate macrophage M2 polarization and be used as the positive control group.^[^ ^27^ ^]^ The results demonstrated that β_Man effectively modulated ECM metabolism, similar to results in NPCs (Figure S6A–G, Supporting Information) and AFCs (Figure S7A–C, Supporting Information). The IF result is the same as the above (FigureS6L–N, S5C and S7G–J, Supporting Information). Meanwhile, the results of Safranin O, Alcian Blue, and β‐gal staining demonstrated that β_Man enhanced collagen production and reduced cellular aging under inflammatory conditions within the co‐culture system (Figures S6H and S7D, Supporting Information). These results demonstrate that β_Man regulates macrophage polarization and protects NPCs/AFCs' function in the co‐culture system.

Inflammation‐driven cellular apoptosis within IVD plays a pivotal role in exacerbating the pathological cascade of IDD.^[^ ^28^ ^]^ FCM and Live/dead staining revealed that β_Man reduced inflammation‐induced apoptosis in both NPCs and AFCs (Figure 4H–J, Figures S3G–H and S8A, Supporting Information). Also, β_Man abrogated M1 polarization‐mediated apoptotic signaling in the compartmentalized co‐culture system, thereby preserving disc cell viability through paracrine pathway modulation (Figures S6I–K, S5B, S7E,F, Supporting Information). JC‐1, commonly used to detect mitochondrial membrane potential, could assess mitochondrial health and the progression of apoptosis. We performed flow cytometry to detect the mitochondrial membrane potential in NPCs (Figure 4K), with CCCP^+^ as the positive control (Figure S8B, Supporting Information). Additionally, we conducted JC‐1staining in NPCs (Figure 4M). The results showed that IL‐1β increased mitochondrial fragmentation, decreased JC‐1 aggregates, and increased monomers. β_Man effectively prevented inflammation‐induced mitochondrial damage (Figure 4R,Figure S8C, Supporting Information).

### 2.5 β_Man Protects NPCs Under Inflammation by Inhibiting Prkcg

**Figure 5:**
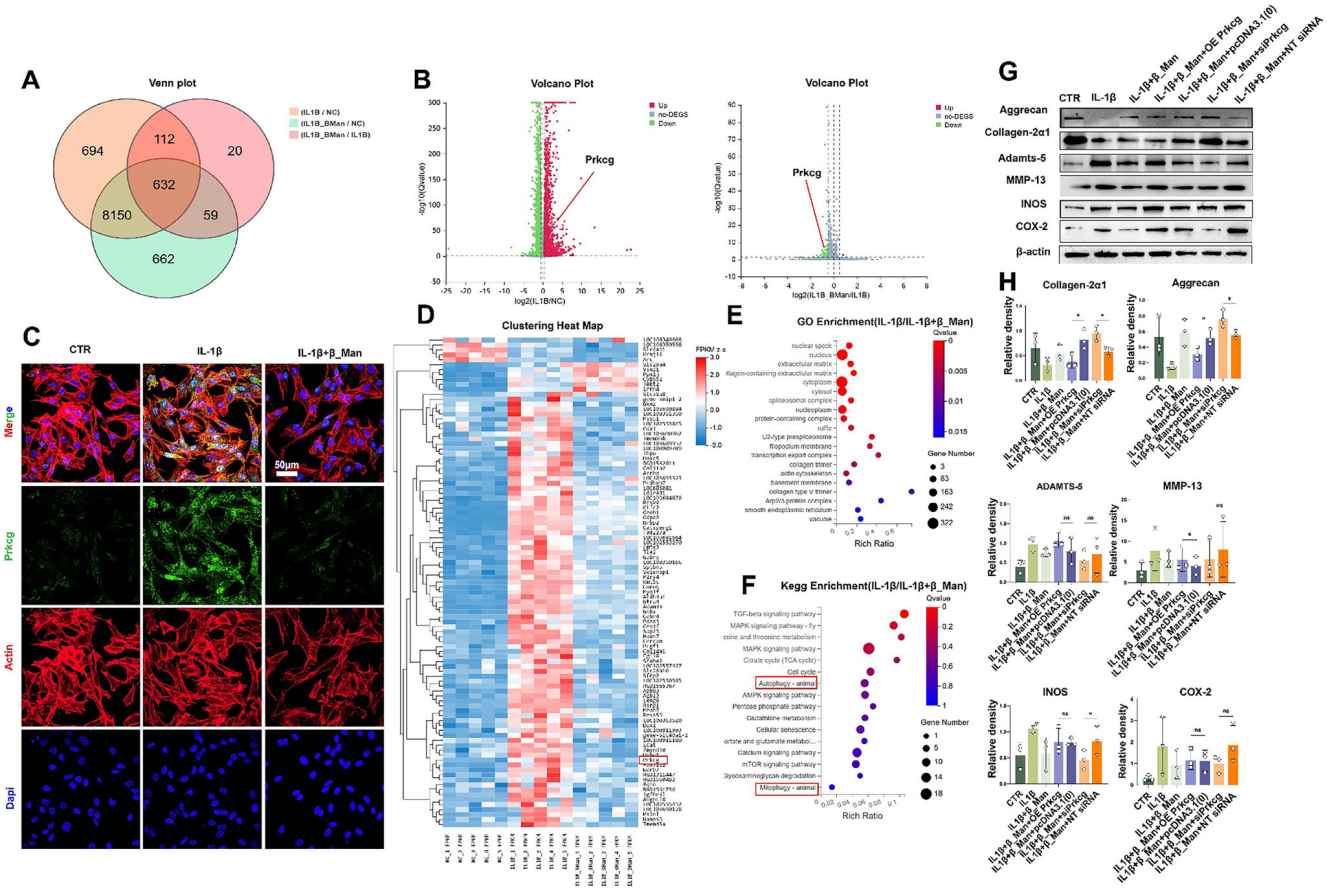
β_Man protects NPCs by inhibiting Prkcg expression. A) Venn diagram showing common gene expression in NPCs with IL‐1β and β_Man (n = 5). B) Hierarchical clustering between the three NPC groups. C) IF of Prkcg in different treatments (n = 3, scale bar: 50 µm). D) The heatmap illustrates differential gene expressions (n = 5). E) GO enrichment analysis of differentially expressed genes. F) KEGG pathway enrichment analysis. G) Western blot analysis of ECM metabolism expression in NPCs under different treatments (IL‐1β, β_Man, siPrkcg, NT siRNA, OE Prkcg, pcDNA3.1(0); (n = 3). H) Quantitative analysis of Western blot. Data is presented as mean ± SD. ∗*p* < 0.05, ∗∗*p* < 0.01, ∗∗∗*p* < 0.001. ns, no significance.

We performed RNA sequencing analysis to investigate the mechanisms and downstream targets of β_Man. These three groups share 632 genes commonly expressed (**Figure** **5**A). Differential gene analysis, including the volcano plot and clustering heatmap (Figure 5B,D), showed upregulation of Prkcg expression in the IL‐1β group compared to the Control (NC) group. Meanwhile, β_Man treatment downregulated Prkcg. Therefore, Prkcg may be a downstream target of β_Man. And protein expression of Prkcg was consistent with the RNA sequencing (Figure 5C, Figure S8D, Supporting Information). Next, we performed silencing (siPrkcg) and overexpression (OE Prkcg) of the Prkcg. NT siRNA and OE pcDNA3.1(0) were used as the negative controls. Western blot was performed to verify the efficiency of the interventions (Figure S8E, Supporting Information). We found that Prkcg silencing significantly enhanced the effect of β_Man, while overexpressing Prkcg significantly weakened the effect of β_Man (Although the results were not statistically significant, obvious trends could still be observed, Figure 5G,H). Finally, we performed KEGG and GO enrichment analyses to explore the biological functions of β_Man in NPCs (Figure 5E,F) and show that β_Man may regulate autophagy in NPCs, which is consistent with previous studies^[^ ^10^ ^]^

### 2.6 Prkcg Knockdown Enhances Mitophagy via the Pink1/Parkin Pathway under Inflammation

**Figure 6:**
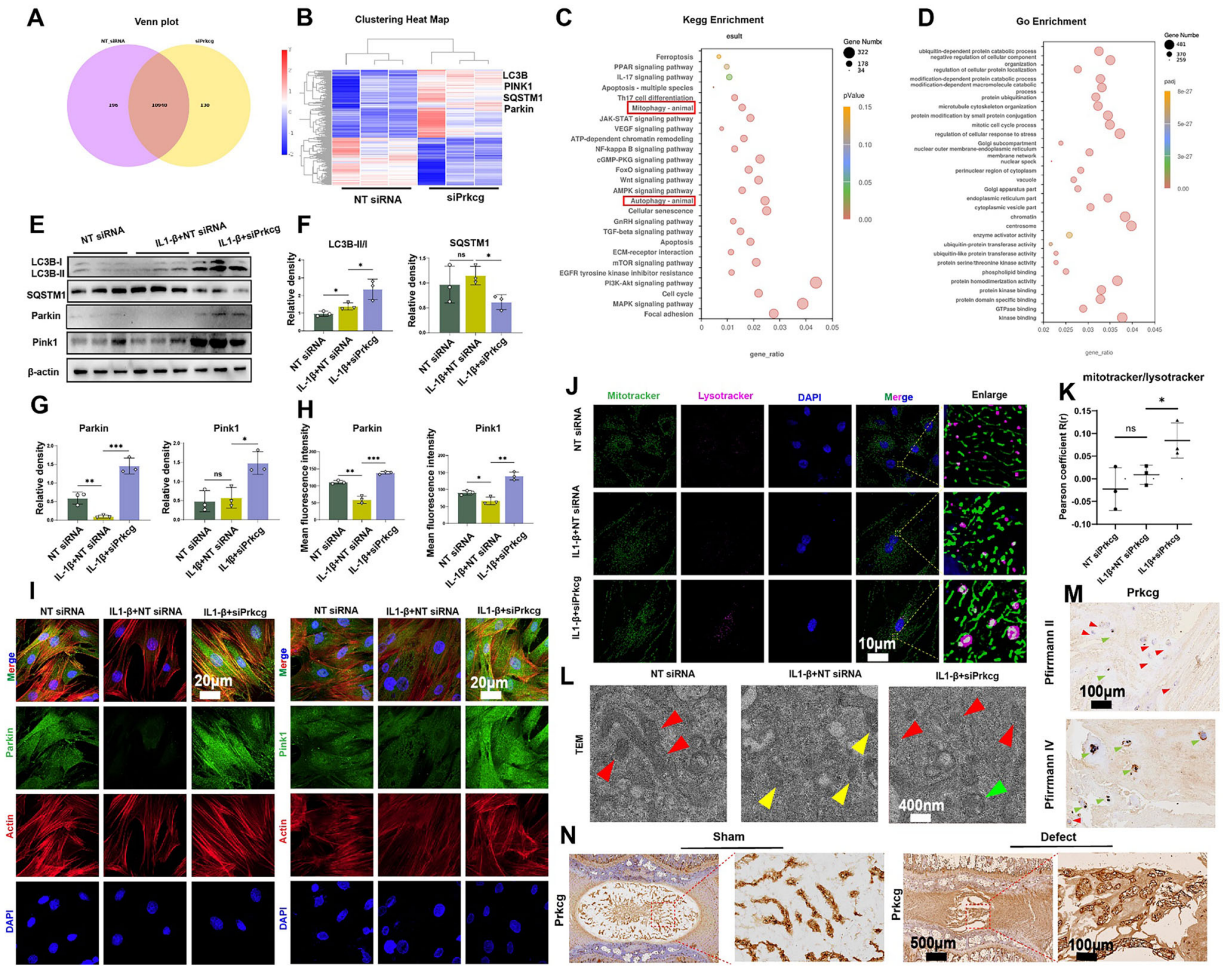
Prkcg knockdown under inflammation enhances mitophagy in NPCs. A) Venn diagram showing common gene expression between NPCs treated with NT siRNA and siPrkcg (n = 3). B) Heatmap of differentially expressed genes (n = 3). C) KEGG enrichment analysis. D) GO enrichment analysis. E) Western blot of LC3B, SQSTM1, Parkin, and Pink1 expression in NPCs treated with NT siRNA, siPrkcg, and IL‐1β (n = 3). F,G) Quantitative analysis of Western blot results. H) Quantitative analysis of IF for Pink1 and Parkin. I) IF of Pink1 and Parkin expression (n = 3, scale bar: 20 µm). J) Colocalization analysis of MitoTracker and LysotTacker (n = 3, scale bar: 10 µm). K) Quantitative analysis of MitoTracker and LysoTracker colocalization. L) TEM of NPCs from different treatment groups (n = 3, scale bar: 400 nm). M) IHC of Prkcg in human NP with Pfirrmann grade II and grade IV (n = 5, scale bar: 100 µm). N) IHC of Prkcg in control and defect groups of IVD in rats (n = 5, scale bar: 500 µm, 100 µm). Data is presented as mean ± SD. ∗*p* < 0.05, ∗∗*p* < 0.01, ∗∗∗*p* < 0.001. ns, no significance.

Previous studies have not explored the role of Prkcg in NPCs. RNA sequencing was conducted to fill this gap, The Venn diagram shows that 10 940 genes are commonly expressed in both groups (**Figure** **6**A). The GO and KEGG enrichment analysis indicate that Prkcg is associated with autophagy and mitophagy (Figure 6C,D). This overlaps with the results of the KEGG analysis of β_Man (Figure 5F). Meanwhile, the clustering heatmap shows that after silencing Prkcg, the expression of autophagy‐related mRNA, including LC3B, SQSTM1, Pink1, and Parkin in NPCs significantly increased (Figure 6B). This indicates that Prkcg may regulate cellular autophagy, and we explored cells in the next. Western blot demonstrated that Prkcg knockdown in NPCs resulted in increased conversion of LC3B‐I to LC3B‐II and a decrease in SQSTM1 following IL‐1β treatment (Figure 6E,F). Western blot and IF results showed that IL‐1β treatment reduced the Pink1 and Parkin, while Prkcg silencing reversed this effect (Figure 6E,G–I). Next, we performed co‐localization staining of NPCs with MitoTracker and LysoTracker to explore the interplay between lysosomes and mitochondria. The results showed that Prkcg silencing increased the interaction between mitochondria and lysosomes under inflammation (Figure 6J,K). Transmission electron microscopy (TEM) showed that the IL‐1β group had more mitochondria with shrinkage, disappearance of mitochondrial cristae, and noticeable pigment deposition (yellow arrows). Prkcg silencing can counteract the inflammatory effects on mitochondria (red arrows), and autophagolysosomes were also observed (green arrows) (Figure 6L). Finally, we collected NP from humans and rats with varying degrees of degeneration and conducted IHC to assess Prkcg trends. The results indicated that Prkcg expression was elevated in advanced stages of degeneration in both rats and humans (Figure 6M,N, Figure S8F, Supporting Information). This demonstrated that Prkcg holds therapeutic potential in humans.

### 2.7 Prkcg Knockdown Effectively Prevents IDD

**Figure 7:**
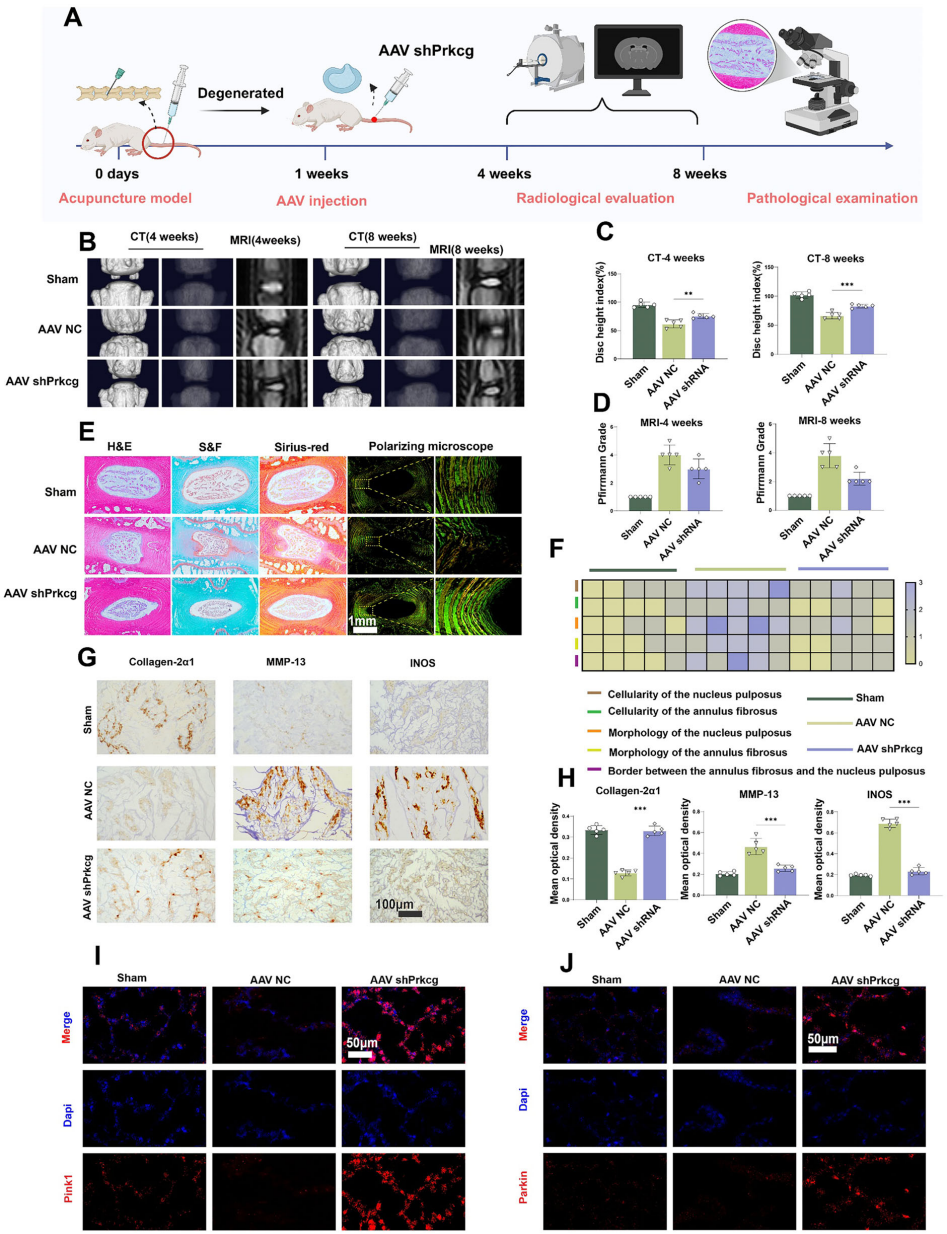
Knockout of Prkcg effectively prevents IDD Progression in vivo. A) Schematic of the experimental workflow in vivo (created using BioRender.com). B) Representative CT and MRI images of rat IVD at 4 and 8 weeks. C) Statistical analysis of CT. D) Statistical analysis of MRI. E) Histological staining (H&E, Safranin O/Fast Green, and Sirius Red combined with polarized microscopy) of IVD (scale bar: 1 mm). F) Heatmap illustrating changes in histological scores G) IHC of Collagen‐2α1, MMP‐13, and INOS (scale bar:100 µm). H) Statistical analysis of IHC. I) IF of Pink1 expression (scale bar: 50 µm). J) IF of Parkin expression (scale bar: 50 µm). Data is presented as mean ± SD, n = 5. *p < 0.05, **p < 0.01, ***p < 0.001. ns, no significance.

We established a Prkcg knockdown model in vivo using adeno‐associated virus‐5 to further evaluate the role of Prkcg in IDD (AAV‐5) (**Figure** **7**A). In brief, 1 week after surgery, AAV‐shPrkcg was injected into the IVD (AAV NC as control). CT and MRI were performed for 4 and 8 weeks (Figure 7B). The results showed that the AAV‐shPrkcg group of rats exhibited better DHI (Figure 7C) and Pfirrmann grading (Figure 7D). At 8 weeks, we performed IVD sectioning and histopathological staining in rats. Techniques such as H&E, Safranin O/Fast Green, and Sirius Red staining combined with a polarized microscope were used to observe the histology of the IVD (Figure 7E). Histological scoring indicated that AAV‐shPrkcg can improve cellular morphology and delay the progression of IDD (Figure 7F). IHC results showed that Prkcg knockdown significantly increased the expression of COL‐2 in the IVD and decreased the expression of MMP‐13 and INOS (Figure 7G,H). Finally, we performed IF to investigate the regulation of autophagy in NP in vivo. The results showed an increase in the expression of Pink1 and Parkin (Figure 7I,J, Figure S9A, Supporting Information), indicating that Prkcg knockdown enhances the Pink1‐Parkin pathway, promoting autophagy.

### 2.8 β_Man Inhibits Prkcg Expression by Reducing TET2‐Mediated DNA Demethylation

**Figure 8:**
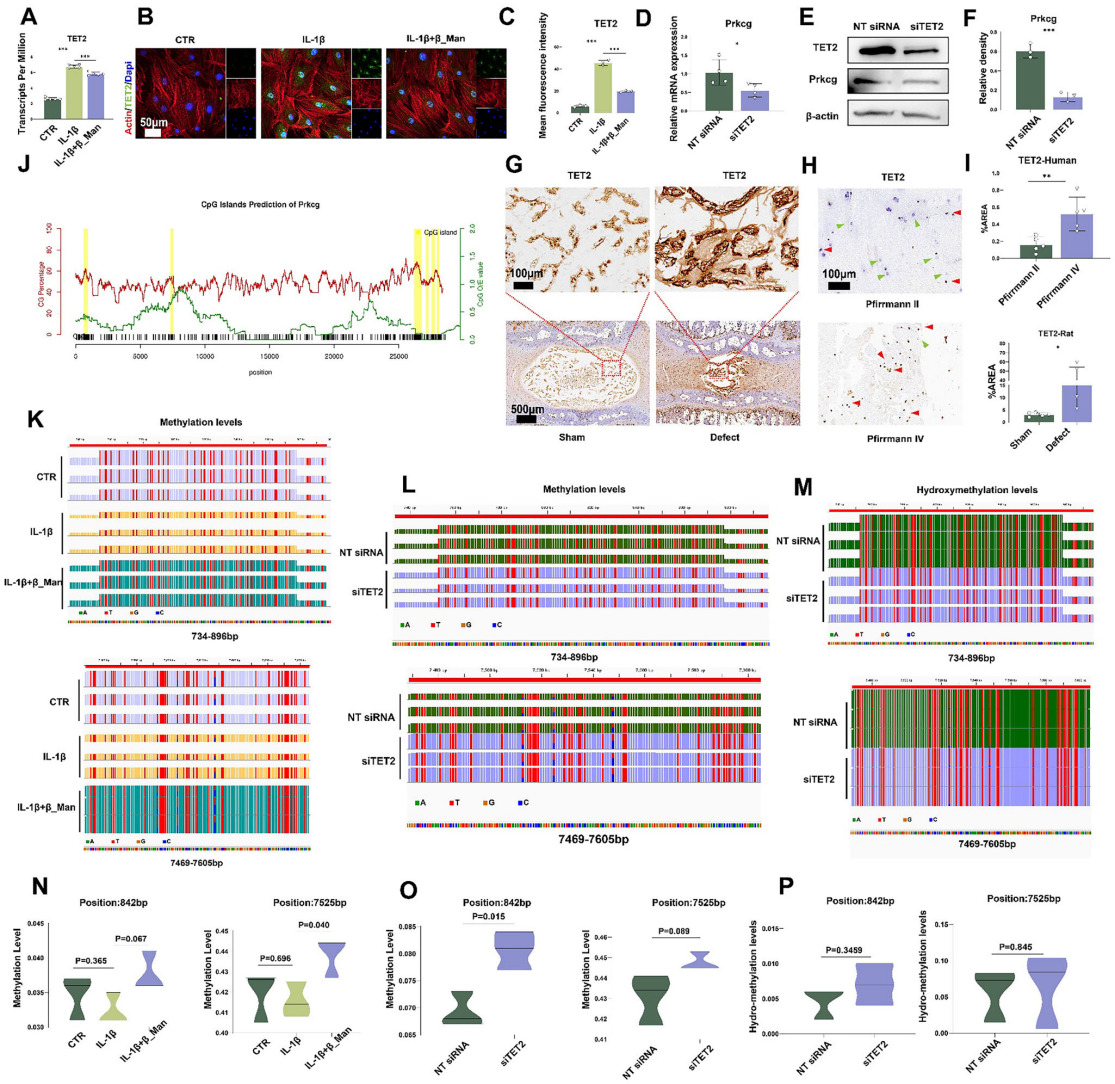
β_Man enhances Prkcg DNA methylation by reducing TET2. A) RNA sequence reveals changes in TET2 (n = 5). B) IF of TET2 expression under different treatments (n = 3, scale bar: 50 µm). C) Quantitative analysis of IF results. D) PCR results showing changes in Prkcg mRNA expression after TET2 silencing (n = 4). E) Western blot analysis of TET2 and Prkcg protein expression following TET2 silencing (n = 3). F) Statistical analysis of Western blot results. G) IHC of TET2 in IVD of rats (n = 5, scale bar: 500 µm, 100 µm). H) IHC of TET2 in human NP (n = 5, scale bar:100 µm). I) Quantitative analysis of IHC results. J) Diagram of Prkcg DNA CpG island locations. K) DMRs of Prkcg in NPCs treated with IL‐1β or β_Man (n = 3). L) DMRs of Prkcg with TET2 silencing (n = 3). M) Differential hydroxymethylation regions (DhMRs) of Prkcg following TET2 silencing (n = 3). N) DMs of Prkcg in NPCs treated with IL‐1β or β_Man (n = 3). O) DMs of Prkcg with TET2 silencing (n = 3). P) Differential hydroxymethylation sites (DhMs) of Prkcg with TET2 silencing (n = 3). Data is presented as mean ± SD. *p < 0.05, **p < 0.01, ***p < 0.001. ns, no significance.

DNA methylation is one of the epigenetic modifications and plays a crucial role in bone and cartilage degeneration. Inflammatory cytokine can increase DNA methylation on IL‐1β promoter in chondrocytes, causing remaining inflammation.^[^ ^29^ ^]^ Decreasing DNA methylation in the promoter regions of certain matrix‐degrading enzymes also enhances the synthesis of these enzymes (such as MMP‐3, MMP‐9, MMP‐13, and ADAMTS‐4), exacerbating the progression of osteoarthritis.^[^ ^30^ ^]^ We analyzed the expression of methylation‐related genes from the RNA sequencing to investigate the epigenetic mechanism underlying β_Man's regulation of Prkcg expression. The results showed that only TET2 was significantly upregulated under IL‐1β treatment (**Figure** **8**A), and β_Man treatment reduced its expression. The expression of other genes (TET1, TET3, Dnmt1, Dnmt3a, Dnmt3b, MECP2, MBD2) have no significant trends (Figure S9B, Supporting Information). IF results demonstrated that the TET2 protein exhibited a consistent trend (Figure 8B,C). TET2, a member of the TET (ten‐eleven translocation) family, is known to be a key regulator of DNA demethylation due to its dioxygenase activity.^[^ ^31^ ^]^ The upregulation of TET2 can effectively promote collagen synthesis and alleviate arthritis.^[^ ^32^ ^]^ However, the role of TET2 in IVD has not yet been clarified. In this study, TET2 was silenced by siTET2 to investigate whether TET2‐mediated DNA methylation regulates the expression of Prkcg. We found that both mRNA and protein of Prkcg were reduced (Figure 8D–F). 5‐methylcytosine (mC) is the most common epigenetic marker, primarily occurring at cytosine‐guanine (CG or CpG) dinucleotides.^[^ ^33^ ^]^ TET enzymes exhibit higher efficiency in oxidizing mC in a CG context.^[^ ^33^, ^34^ ^]^ We performed Targeted Bisulfite Sequencing (TBS) on the CpG island region of the Prkcg (Figure 8J). The results showed significant differential methylation in two differentially methylated regions (DMRs), 734–896 and 7469–7605 bp. IL‐1β led to a notable decrease in methylation. At the same time, β_Man enhanced the methylation in those DMRs (Figure 8K). Also, TET2 silencing significantly enhanced the methylation in the two DMRs compared to the control group (Figure 8L). We further examined the methylation status of specific differential methylation sites (DMs). β_Man treatment and TET2 silencing led to an increasing trend in the methylation of two DMs, 842 and 7525 bp (Figure 8N,O). The oxidation of 5‐mC to 5‐hydroxymethylcytosine (5‐hmC) is a common process in TET2‐mediated demethylation.^[^ ^33^ ^]^ We examined the hydroxymethylation level of Prkcg after TET2 silencing through Targeted Ace Sequencing (TAS). However, no significant differences were observed in the aforementioned regions and sites (Figure 8M,P). In conclusion, these results indicate that β_Man reduces the expression of TET2, decreases TET2‐mediated demethylation, and increases the DNA methylation of Prkcg in an inflammatory environment. Finally, we collected NP samples from rats (Figure 8G) and humans (Figure 8H) with different degrees of degeneration. The IHC results showed that TET2 expression significantly increased with the severity of degeneration (Figure 8I).

### 2.9 Overexpression of TET2 Attenuated the Effect of β_Man in Preventing IDD

**Figure 9:**
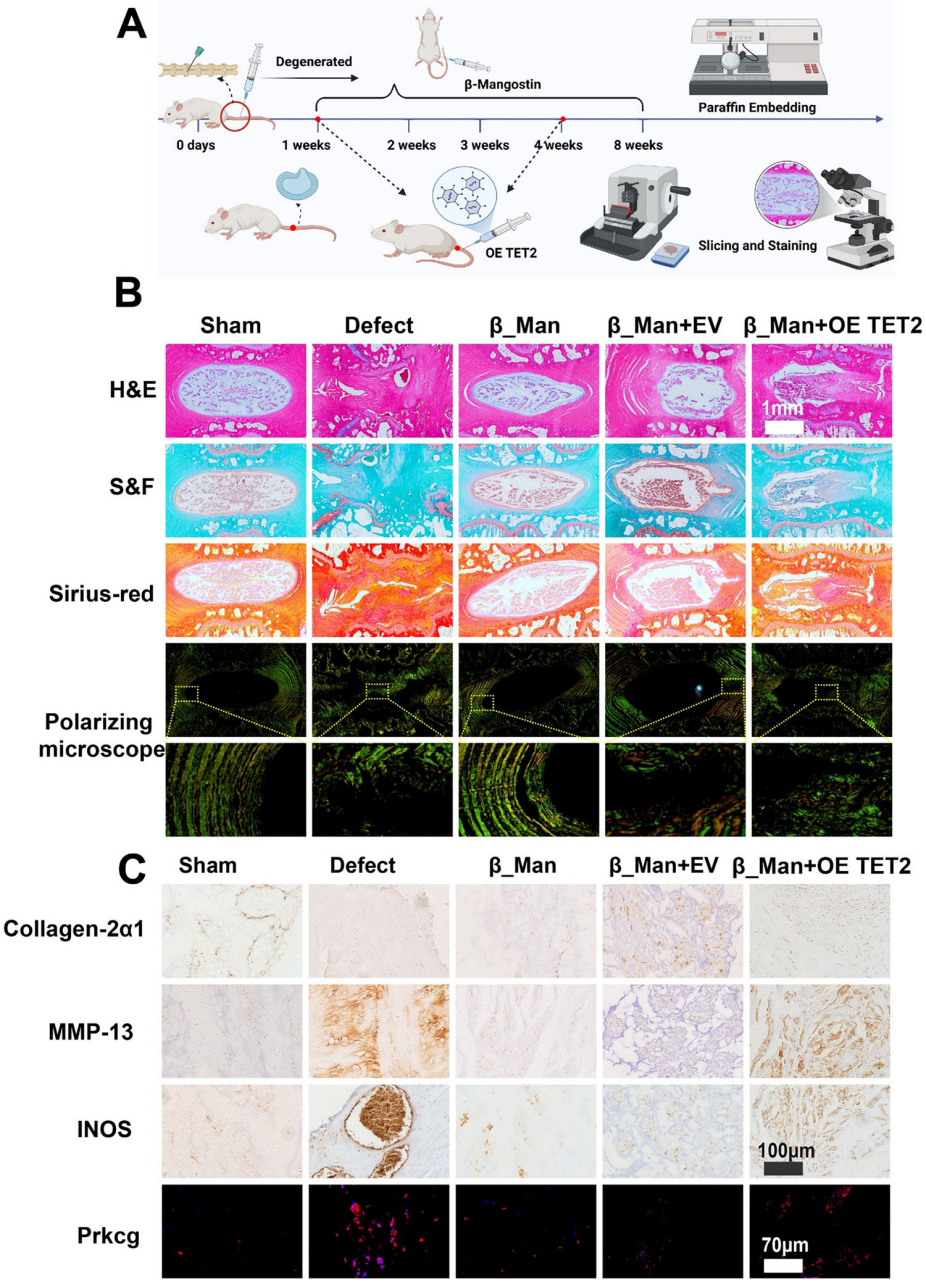
Overexpression of TET2 attenuated the effect of β_Man in preventing IDD. A) Schematic of the in vivo experimental workflow (created using BioRender.com). B) Histological analysis of IVD (n = 5, scale bar:1 mm). C) IHC and IF of Collagen‐2α1, MMP‐13, INOS, and Prkcg in different groups of rats (n = 5, scale bar: 100 µm, 70 µm).

To elucidate the functional impact of TET2 on IVD homeostasis, we engineered TET2‐overexpressing NPCs through plasmid transfection. Subsequent Western blot analysis of ECM metabolic markers revealed two pivotal observations: First, TET2 overexpression significantly upregulated Prkcg expression, a finding consistent with our prior mechanistic studies. Second, and more critically, we detected a coordinated shift in ECM dynamics characterized by suppressed synthesis markers alongside elevated degradation enzymes and pro‐inflammatory mediators. These reciprocal alterations provide compelling evidence that TET2 overexpression disrupts ECM metabolic equilibrium in NPCs (Figure S10A,B, Supporting Information). Subsequently, we constructed a TET2‐bound adenovirus to investigate whether TET2 further influences the therapeutic effects of β_Man in vivo (**Figure** **9**A). We observed that compared to the EV (Empty Vector) group, overexpression of TET2 reduced the sensitivity of rat IVD to the therapeutic effects of β_Man. Histological analysis revealed that the IVD in the TET2 overexpression group exhibited irregular morphology. There was partial replacement of the NP by fibrous cord‐like tissue, with indistinct boundaries between the NP and the AF, as well as abnormal cellular structures within (Figure 9B, Figure S10C, Supporting Information). The IHC showed that the group of TET2 overexpression exhibited increased expression of COL‐2α1 and decreased expression of MMP‐13 and INOS (Although no significant statistical differences in INOS expression were observed within the group, a clear upward trend was still evident) (Figure 9C, Figure S10D, Supporting Information). Finally, we conducted IF to detect the effect of TET2 overexpression on Prkcg. The results showed that the expression of Prkcg increased significantly in the defect group, and β_Man could effectively reduce the expression of Prkcg. Meanwhile, TET2 overexpression inhibited the inhibitory effect of β_Man on Prkcg (Figure 9C, Figure S10D, Supporting Information). These results indicated that β_Man inhibits IDD progression through TET2‐Prkcg.

## 3 Discussion

**Figure 10:**
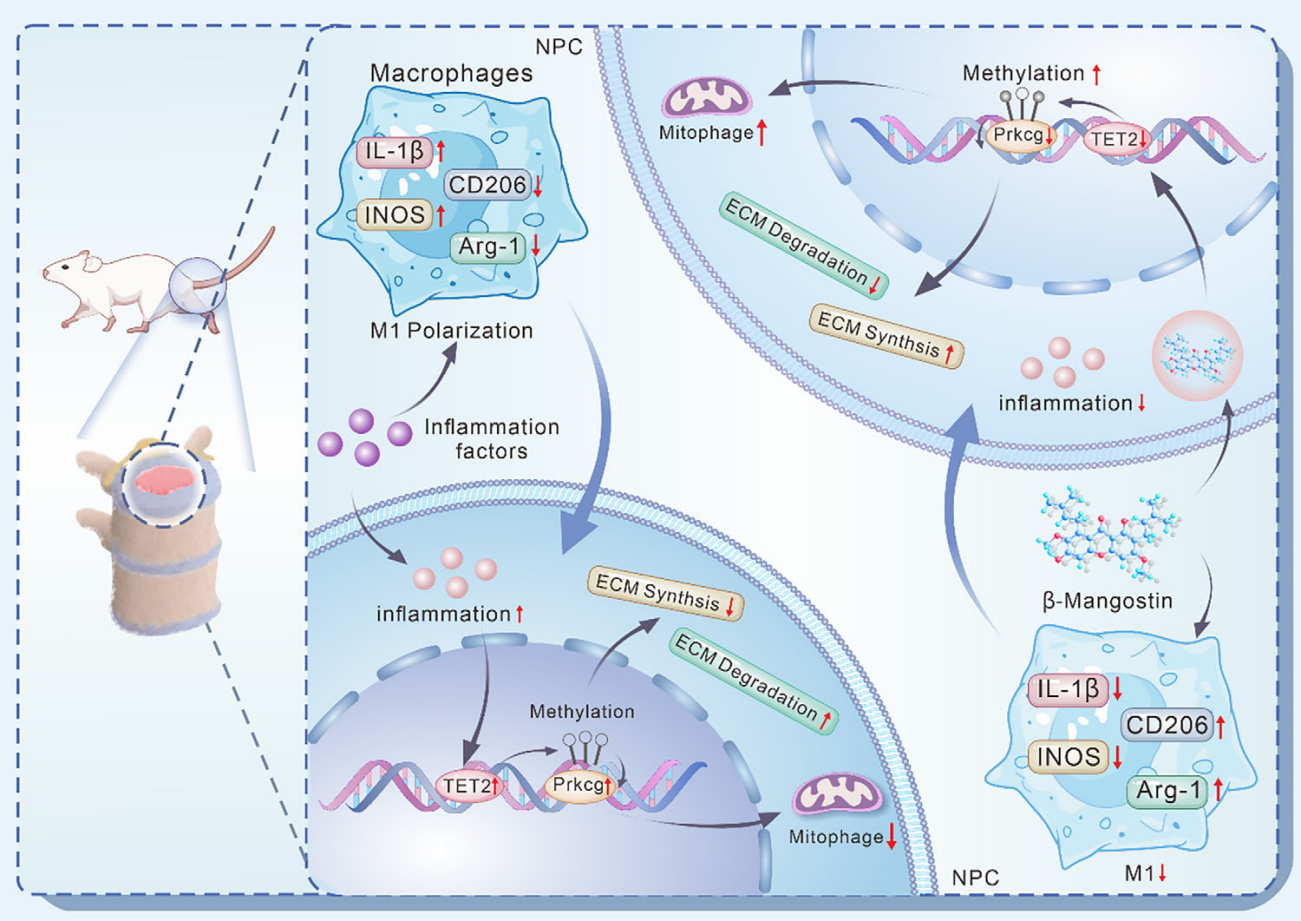
Schematic representation of the mechanisms that β_Man therapeutic effects in treating IDD. β_Man inhibits M1 polarization of macrophages and improves ECM metabolism disorder of NPCs by inflammation‐induced. In NPCs, β_Man mitigates inflammation‐induced inhibition of Prkcg methylation by suppressing TET2‐mediated demethylation, which decreases Prkcg and increases mitophagy of NPCs and inhibits the progression of IDD.

The incidence of symptomatic lumbar disc herniation in patients with IDD is1%–3%. The clinical manifestations mainly include pain, muscle spasms, restricted mobility, and sensory or motor neurological deficits.^[^ ^35^ ^]^ Clinically, IDD is primarily managed with palliative treatments, including pain‐relief medications, acupuncture, physical therapy, or surgical removal of the herniated disc.^[^ ^36^ ^]^ These methods aim to alleviate symptoms and prevent the condition from affecting normal daily activities; however, they cannot fundamentally halt IDD progression. Thus, exploring biological therapies and understanding the mechanisms underlying IDD degeneration to delay or reverse its progression from the source has become the main focus of the present research. AF develops into microtears with age and IDD progression, aiding the ingrowth of blood vessels and nerves. M1 macrophages progressively accumulate within the disc,^[^ ^37^ ^]^ releasing proinflammatory cytokines.^[^ ^38^ ^]^ and exacerbating IDD. Herein, we found that β_Man effectively suppressed M1 polarization of macrophages, reduced inflammation, directly or indirectly alleviated inflammation‐induced dysfunction of ECM synthesis and prevented cell apoptosis in NPCs and AFCs. These findings highlight the β_Man's considerable potential in treating IDD (**Figure** **10**).

Serine/threonine‐specific protein kinases (STKs) are a family of kinases that can phosphorylate the hydroxyl (OH) group on the serine or threonine residues of proteins, thereby playing a critical role in regulating different cellular activities.^[^ ^39^ ^]^ Notably, star members of the STKs family, such as MAPK and AKT, have become focal points of research because of their involvement in inflammation regulation, cell cycle, oxidative stress, and different cellular functions.^[^ ^40^ ^]^ STKs contribute to IDD alleviation,^[^ ^41^ ^]^ and interact with the mTOR pathway, modulating processes such as autophagy and mitophagy,^[^ ^42^ ^]^ in bone and cartilage. Prkcg, a member of the protein kinase C family, belongs to the broader STK category. Previous studies predominantly focused on their roles in the nervous system, including neurotransmitter release, cell proliferation, and differentiation.^[^ ^20^, ^43^ ^]^ Increased Prkcg expression has been associated with mechanical allodynia after nerve injury,^[^ ^44^ ^]^ and spinocerebellar ataxia.^[^ ^45^ ^]^ Furthermore, Prkcg is involved in tumor regulation and growth.^[^ ^46^ ^]^ Prkcg has been previously suggested to be specifically expressed in the nervous system.^[^ ^14^, ^47^ ^]^ However, we confirmed its presence in NPCs at mRNA and protein levels, where it exerts a critical function. We identified Prkcg as a potential downstream target of β_Man based on RNA sequencing data. β_Man mitigates ECM metabolic dysregulation by inhibiting the inflammation‐induced Prkcg upregulation. Simultaneously, Prkcg knockdown effectively alleviated local inflammation in the IVD and prevented IDD progression in vivo. Additional analysis of NP from Pfirrmann grade 4 showed Prkcg overexpression compared to grade 2. In rats, the defect group demonstrated substantially higher levels than the control group. These findings underscore Prkcg differential expression across the NP degeneration stages, suggesting its potential as a preclinical diagnostic marker and therapeutic target for IDD.

Autophagy is a conserved degradation process that regulates the removal of dysfunctional cellular components via a lysosome‐dependent mechanism.^[^ ^48^ ^]^ Mitochondria, as a critical source of intracellular energy, are necessary for regulating redox balance and orchestrating cellular responses to stress.^[^ ^49^ ^]^ NPCs’ survival, proliferation, and ECM metabolism primarily depend on a healthy mitochondrial quality control system regulated by mechanisms of mitochondrial monitoring.^[^ ^50^ ^]^ Mitophagy is a specialized form of selective autophagy that degrades damaged mitochondria and maintains mitochondrial homeostasis.^[^ ^51^ ^]^ Although IVD is a hypoxic environment and NPCs mainly rely on glycolysis to produce energy,^[^ ^52^ ^]^ they still have a functional mitochondrial network that allows active mitochondrial flux to adjust mitochondrial numbers as per metabolic demand.^[^ ^53^ ^]^ Defective mitophagy accelerates IDD progression.^[^ ^54^ ^]^ Prkcg, a downstream target of β_Man, was found to be pivotal in mitophagy. SQSTM1 binds to LC3B on the mitochondrial membrane to form autophagosomes, which subsequently fuse with lysosomes, triggering autophagic flux and accelerating protein aggregate clearance.^[^ ^55^ ^]^ The increase in the conversion of LC3B‐I to LC3B‐II and the decrease in SQSTM1 indicated smooth autophagic flux, reflecting increased autophagic activity and efficient clearance of damaged cellular components. The PINK1 axis (also a serine/threonine kinase) and the E3 ubiquitin ligase Parkin are necessary for increasing mitophagy. PINK1's kinase activity is a prerequisite for activating Parkin translocation to depolarized mitochondria, thereby initiating mitophagy.^[^ ^56^ ^]^ Here, Prkcg knockdown led to elevated SQSTM1 mRNA levels, implying that autophagic flux may be restrained. However, Prkcg knockdown resulted in decreased SQSTM1, enhanced LC3B‐I conversion to LC3B‐II, and upregulation of Pink1 and Parkin expression following IL‐1β treatment. This shows that inflammation may serve as a main regulatory factor in mitophagy. However, further investigation is required to explore this mechanism. In conclusion, Prkcg plays a crucial role in preventing IDD progression. Its downregulation effectively enhances mitophagy in NPCs, protects ECM metabolism, and promotes IVD repair. This implies that the inflammatory microenvironment is a key mitophagy regulator. Prkcg downregulation effectively increases mitophagy in NPCs, protects ECM metabolism, and promotes IVD repair during inflammation.

DNA methylation is an epigenetic modification that regulates gene expression without changing the genomic sequence.^[^ ^57^ ^]^ The main form of DNA modification is 5‐methylcytosine (5mc), which is crucial in development and disease as it represses transposable elements and regulates transcription.^[^ ^58^ ^]^ TET, a member of the family of Fe^2+^‐ and 2‐oxoglutarate‐dependent dioxygenases, oxidizes 5mC in DNA to 5‐hydroxymethylcytosine (5hmC), which undergoes further oxidation to form 5‐formylcytosine (5fC) and 5‐carboxylcytosine (5caC). These are key intermediates in DNA demethylation's active process.^[^ ^59^ ^]^ We noted that β_Man suppresses the inflammation‐induced overexpression of TET2 in NPCs, which reduces Prkcg demethylation. The sites of potential methylation regulation are at 842 and 7525 bp, and the methylation levels of Prkcg at these two DMs increased following TET2 knockdown. However, the TET2 knockdown did not substantially decrease the conversion of 5mC to 5hmC. This implies that TET2 regulation of Prkcg DNA methylation does not directly reduce 5mC conversion to 5hmC or that 5hmC is quickly oxidized in the subsequent steps.

Thus, β_Man downregulates TET2 expression, resulting in an elevation in Prkcg DNA methylation level, which subsequently decreases Prkcg expression. This process alleviates ECM metabolic disorders in NPCs, increases mitophagy, protects NPCs’ function, and promotes disc repair.

This study has a limitation. Although we have explored the regulatory mechanism of a “novel gene” Prkcg in the context of IDD, we have only examined the regulation of its DNA methylation and predicted the involved CpG sites, without conducting targeted verification of these sites. Additionally, TET2 oxidizes 5mC to 5hmC.^[^ ^59^ ^]^ We did not validate the other two oxidation products, 5fC and 5caC; however, owing to technical limitations, we could not measure the transformation of these substances. Fortunately, this does not hinder the elucidation of β_Man's mechanism. Finally, we did not use Prkcg‐knockout mice to confirm these effects. These issues need further investigation.

## 4 Conclusion

We describe the mechanisms through β_Man exerts its therapeutic effects in the treatment of IDD, and demonstrate that it inhibits M1 macrophage polarization, improves ECM metabolic dysregulation in NPCs and AFCS, prevents cellular senescence and apoptosis, and identifies its potential downstream target, Prkcg. Next, we further elucidate the regulatory mechanism of β_Man. Prkcg, as a downstream target, is regulated through methylation mediated by TET2. β_Man silences TET2, leading to increased Prkcg methylation and subsequent knockdown Prkcg. which alleviates local inflammation in IVDs, enhances mitophagy, and prevents the progression of IDD. Prkcg may serve as a preclinical diagnostic biomarker and therapeutic target for IVDD.

## 5 Experimental Section

### Cell Isolation, Culture, and Seeding

The NPCs and AFCs used in this study were extracted from 4‐week‐old male Sprague‐Dawley rats (Vital River, China). After euthanizing the rats using excessive carbon dioxide, the bodies were disinfected with 75% ethanol for 30 min. IVD was excised using surgical scissors, and the NP and AF were separated. The collected tissues were enzymatically digested in 0.25% trypsin for 30 min. Subsequently, the NP was digested with 0.2% collagenase II (Solarbio, China), and the AF was digested with a mixture of 0.2% collagenase I and 0.2% collagenase II (Solarbio, China) for 4 h. After centrifugation, the cell pellets were collected and washed twice with PBS. The NPCs and AFCs were cultured in DMEM/F‐12 medium supplemented with 10% fetal bovine serum (FBS; Servicebio, China), 100 µg mL^−1^ penicillin, and 100 µg mL^−1^ streptomycin (Servicebio, China). The culture medium was refreshed every 2 days to maintain cell growth. Once the adherent cells reached 80–90% confluence, they were passaged. Trypsin‐EDTA solution (0.25%) was added to the culture dishes to digest the cells for 1 min, followed by adding a fresh medium to terminate the digestion. The resulting cell suspension was centrifuged, and the cell pellet was resuspended in a fresh medium for further subculturing. In this study, cells within three passages were used for the experiments. For in vitro assays, cells were stimulated with IL‐1β (Abclonal, China) for 24 h to simulate an inflammatory environment. Subsequently, β_Man (MCE, USA) and TGF‐β (Abclonal, China) were added for treatment, followed by further experiments.

The macrophages used in this study were extracted from 4‐week‐old male Sprague‐Dawley rats. After euthanizing the rats with excess carbon dioxide, the bodies were disinfected with 75% ethanol for 15 min. Using surgical scissors, the intact femur and tibia were extracted, and the bone ends were removed. The bones were placed in a sterile culture dish, and the bone marrow cavity was repeatedly flushed with DMEM. The collected bone marrow was transferred to DMEM medium supplemented with 10% FBS, 100 µg mL^−1^ penicillin, and 100 µg mL^−1^ streptomycin, and cultured at 37 °C in a 5% CO₂ incubator. After 24 h, non‐adherent cells were collected and resuspended in fresh DMEM medium containing 30 ng mL^−1^ M‐CSF (Abclonal, China), 10% FBS, 100 U mL penicillin, and 100 µg mL streptomycin. The cells were cultured until adherence before proceeding with subsequent experiments. LPS (100 ng mL^−1^, Abclonal, China) was used to induce M1 macrophage polarization.

For cell co‐culture, macrophages were cultured in Transwell inserts (Corning, USA) and co‐cultured with NPCs (or AFCs) seeded in the lower chamber at a ratio of NPCs to macrophages of 1:1. All cultures were maintained at 37 °C in a 5% CO₂ incubator. All experimental protocols were approved by the Ethics Committee of Qilu Hospital, Shandong University.

### Cell Viability CCK‐8 Assay

Cell viability changes were measured using the Cell Counting Kit‐8 (CCK8) assay. Cells were seeded into 96‐well plates and cultured at 37 °C. After adding different concentrations of β_Man, cells were cultured for 24 or 48 h. Then, 10 µL of CCK8 reagent (Beyotime, China) was added to each well, and the cells were further incubated at 37 °C for 2 h. The optical density (OD450) value was measured using a Multiskan enzyme reader (Infinite E Plex, Tecan, Switzerland). The experiment was repeated at least three times.

### Quantitative Reverse Transcriptase‐Polymerase Chain Reaction

Total RNA was extracted from cells or tissues using Triazole reagent (Invitrogen, USA) according to the manufacturer's instructions. RNA concentration was quantified using a NanoDrop spectrophotometer (Thermo Fisher Scientific, MA, USA). Then, cDNA was synthesized using the Hifair AdvanceFast first Strand cDNA Synthesis SuperMix for qPCR (DNA digester plus) (Yeasen, China) following the manufacturer's protocol.

Subsequently, qPCR was performed using the Hieff qPCR SYBR Green Master Mix (No Rox) (Yeasen, China) in a qTOWER3G (Jena, Germany). The cycle threshold (Ct) values, representing the number of cycles required to reach the threshold fluorescence intensity, were determined from the amplification curves. The mRNA levels were normalized to GAPDH (human) or β‐Actin (Rat) mRNA levels, and the relative expression of the target gene was calculated using the 2^−ΔΔCt^ method. The primer sequences are listed in Tables S3 and S4, Supporting Information.

### Protein Extraction and Western Blot

After cell culture and treatment, cells were lysed for 30 min using RIPA lysis buffer (Beyotime, China) containing protease inhibitors (Thermo Fisher, USA). The samples were then centrifuged at 13 000 rpm for 30 min at 4 °C to collect the supernatant. Protein concentration was measured using the BCA protein assay kit (Beyotime, China).

The isolated proteins were separated by sodium dodecyl sulfate‐polyacrylamide gel electrophoresis (SDS‐PAGE) and transferred onto a PVDF membrane (Millipore, USA). The membrane was then blocked with 5% milk for 1 h.

The membrane was then incubated with the anti‐β‐actin (1:50 000, AC026, Abclonal, China), anti‐Collagen2α1(1:500, A19308, Abclonal, China), anti‐Aggrecan (1:1000, A8536, Abclonal, China), anti‐MMP13 (1:1000, A1606, Abclonal, China), anti‐ADAMTS5 (1:1000, A2836, abalone, China), anti‐INOS (1:1000, 18985‐1‐AP, Proteintech, China), anti‐COX‐2 (1:1000, 12375‐1‐AP, Proteintech, China), anti‐Prkcg (1:1000, bs3626R, Bioss, China), anti‐TET2 (1:1000, a5682, Abclonal, China), anti‐Pink1 (1:1000, 23274‐1‐AP, Proteintech, China), anti‐Parkin (1:1000, 14060‐1‐AP, Proteintech, China), anti‐LC3B (1:1000, 14600‐1‐AP, Proteintech, China), anti‐SQSTM1 (1:1000, 18420‐1‐AP, Proteintech, China) overnight. The membrane was then incubated with HRP‐conjugated Goat anti‐Rabbit IgG (1:2000, Abclonal, AS014, China) for 1 h. The membranes were visualized using ECL Plus (Thermo Fisher Scientific, USA). The membrane density from three independent experiments was quantified using ImageJ (National Institute of Health, Bethesda, USA), and the protein levels were normalized to β‐actin.

### Flow Cytometry

The cells were collected and washed twice with ice‐cold PBS, then resuspended in 100 µL of PBS to obtain a single‐cell suspension. Apoptosis was detected using Annexin V/PI staining (C1062S, Beyotime, China), mitochondrial membrane potential was assessed using the Mitochondrial Membrane Potential Assay Kit with JC‐1 (M8650, Solarbio, China), and macrophage polarization was analyzed using CD11b, CD206, and CD86 as markers. After staining for 1 h, cells were washed twice with PBS and resuspended in 300 µL PBS. The cell suspension was analyzed using the BD FACS Celesta Flow Cytometer (BD Biosciences, USA).

### Live/Dead Cell Staining

Cells were seeded on 6‐well plates. After the cells adhered to the plate, stimulants were added. 2 days later, the culture medium was aspirated, and the cells were washed 1–2 times with PBS to remove any non‐adherent cells (gentle washing was performed to avoid detaching the adherent cells). The cells were then stained using the Calcein/PI Cell Viability/Cytotoxicity Assay Kit (Beyotime, China). After incubation at 37 °C in the dark for 30 min, fluorescence images were captured using a fluorescence microscope (Nikon, Ti2‐U, Japan). Green fluorescence indicates live cells, while red fluorescence indicates apoptotic cells.

### IF Staining (Cell)

Seed the cells onto cell culture slides in a 6‐well plate and treated them according to the specified conditions for the designated number of days. After the treatment, remove the culture medium and wash the cells three times with PBS. Add 500 µL of 4% paraformaldehyde to each well and fix the cells for 20 min. Then, add 500 µL of 0.5% Triton X‐100 (Sigma, USA) to each well to permeabilize the cells for 10 min. Afterward, add 1 mL of 5% BSA (Sigma, USA) to each well and block the cells for 1 h. Add anti‐Collagen‐2α1 (1:100, A19308, Abclonal, China), anti‐MMP‐13 (1:100, A1606, Abclonal, China), anti‐INOS (1:100, 18985‐1‐AP, Proteintech, China), anti‐Pink1 (1:200, 23274‐1‐AP, Proteintech, China), anti‐Parkin (1:100, 14060‐1‐AP, Proteintech, China), anti‐Prkcg (1:100, bs3626R, Bioss, China), anti‐TET2 (1:100, a5682, Abclonal, China). to each well and incubate overnight at 4 °C in the dark. Then, add FITC‐conjugated goat anti‐rabbit secondary antibody (1:100, Solarbio, China) and incubate in the dark at room temperature for 1 h.

Wash the cells three times with PBS, then stain the cytoskeleton with Rhodamine‐labeled phalloidin (1:200, Solarbio, China) for 1 h. Finally, wash the cells with PBS three times. Then mount the cell coverslip on a glass slide using an antifading mounting medium (with DAPI, S2110, Solarbio, China). Capture images using a fluorescence microscope (Nikon, Ti2‐U, Japan) or a confocal microscope (Olympus SpinSR10, Olympus, Japan).

For macrophage polarization, IF using the TSA method, briefly, after fixing the macrophages, add citrate buffer (pH 6.0) to the wells, then place the entire plate in a microwave oven and heat at high power for 30 s. Allow the plate to cool to room temperature. Add Triton X‐100 (Solarbio, China) at room temperature for membrane permeabilization for 20 min. Then, add 10% goat serum and incubate at room temperature for 30 min. Following that, add CD86 (1:200, ER1906‐01, rabbit monoclonal, HuaBio) and incubate overnight at 4 °C. Add anti‐rabbit IgG (H+L) AB HRP (1:400, SeraCare, USA) to cover the coverslip and incubate at room temperature for 50 min in the dark. Then, add tyramide salt‐CY3 and incubate at room temperature for 20 min. Afterward, add 1 × citrate repair solution to the well and perform antigen retrieval by heating it in the microwave on high for 30 s, then allow it to cool naturally to room temperature. Add 10% goat serum to the coverslip and incubate at room temperature for 10 min. Then, add CD206 (1:500, ab300621, rabbit monoclonal, Abcam) and incubate overnight at 4 °C. Add ANTI‐RABBIT IGG (H+L) AB HRP (1:400, SeraCare, USA) to cover the coverslip and incubate in the dark at room temperature for 50 min. Then, add tyramide salt‐488 and incubate at room temperature for 20 min. Stain the cell nuclei with DAPI (Solarbio, China). Capture images using a fluorescence microscope.

### IF Staining (IVD)

After dewaxing and antigen retrieval, the slides were blocked with 3% BSA for 30 min. Then, the slides were incubated with anti‐Pink1 (1:200, 23274‐1‐AP, Proteintech, China), anti‐Parkin (1:100, 14060‐1‐AP, Proteintech, China) and anti‐Prkcg (1:100, bs3626R, Bioss, China) overnight at 4 °C. Afterward, the slides were incubated with CY3‐conjugated goat anti‐rabbit antibody (1:300, GB21303, Servicebio, China) in the dark at room temperature for 50 min, followed by DAPI (C0065, Solarbio, China) staining for 5 min to label the cell nuclei. The slides were then mounted using an anti‐fluorescence quenching mounting medium and scanned with a VS200 (Olympus, Japan). Fluorescence intensity was quantified using ImageJ.

### IF Staining (NP Samples of Human)

The paraffin sections were dewaxed to water, followed by antigen retrieval using a citric acid repair solution. Then the sections were blocked with 10% BSA for 30 min. The sections were incubated with the anti‐Aggrecan (1:200, GB11373, Servicebio, China), annti‐CD86 (1:200, HUABIO, China), anti‐CD206(1:500, ab300621, Abcam, Britain), anti‐CD11b (1:1000, ab133357, Abcam, Britain) overnight at 4 °C. Subsequently, the sections were incubated with the secondary antibody for 50 min, and the Tyramide Signal Amplification (TSA) reagent was then applied to the sections. The above steps were repeated three times, followed by counterstaining of the nuclei with DAPI. After mounting, images were captured using a fluorescence microscope (Olympus, Japan).

### JC‐1 Staining

According to the JC‐1 fluorescent probe kit (Beyotime, China). Prepare the working solution and add it to the wells. Stain the cell nuclei with Hoechst (Solarbio, China) and incubate at 37 °C for 30 min. Capture images using a confocal microscope (Olympus SpinSR10, Olympus, Japan). Under fluorescence microscopy, when the mitochondrial membrane potential is high, JC‐1 accumulates in the mitochondrial matrix and forms aggregates, emitting red fluorescence. When the mitochondrial membrane potential is lower, JC‐1 does not accumulate in the mitochondrial matrix and remains in its monomer form, emitting green fluorescence. Statistical analysis of the fluorescence intensity ratio is performed to evaluate the JC‐1 results.

### Safranine O and Alcian Blue Staining

The cells to be detected were fixed with 4% paraformaldehyde at room temperature for 20 min and then incubated with Safranine O or Alcian Blue solution (Solarbio, China) at room temperature for 30 min. After washing three times with PBS, images were captured under a microscope (Olympus, Tokyo, Japan).

### SA‐β‐Gal Staining

The cells to be detected were washed with PBS, and then 1 mL of β‐galactosidase staining fixative was added for fixation at room temperature for 10 min. After that, a staining working solution (Beyotime, China) was added for 2 h. After washing three times with PBS, images were captured under a microscope (Olympus, Tokyo, Japan).

### Mitochondrial Lysosome Co‐Localization Staining

Mitochondria were labeled using Mitotracker (Beyotime, China), and lysosomes were labeled using Lysotracker (Beyotime, China). After imaging, the Pearson correlation coefficient R was calculated to represent the co‐localization correlation between mitochondria and lysosomes.

### TEM

The nucleus pulposus cells were fixed with 4% paraformaldehyde fixative. The samples were then post‐fixed, dehydrated, and embedded. Ultrathin sections were obtained using a Reichert ultramicrotome (Reichert‐Jung, Germany). The stained sections were observed under a field emission electron microscope (Thermo Fisher Scientific, USA). The sections were stained with saturated uranyl acetate‐lead citrate and observed under the field emission transmission electron microscope (Thermo Fisher Scientific, USA).

### Cell Transfection Experiment

The siRNAs targeting rat Prkcg and TET2, as well as the Prkcg overexpression plasmid, were designed and synthesized by Gene&Bio Co. Ltd (China). According to the manufacturer's protocol, Lipofectamine 3000 transfection reagent (Invitrogen) was used for transfection. Cells were seeded in culture dishes for 24 h. Small interfering RNA (siPrkcg, siTET2), overexpression plasmid (OE Prkcg), and the corresponding control RNA (siRNA NC, pcDNA 3.1(0)) were transfected into the cells during the logarithmic growth phase, when the cells were at 70%–80% confluence. After 8 h of transfection, the medium was replaced, and further treatments were applied. Western blot was used to assess transfection efficiency. The siRNA sequences are provided in SI Appendix, data.

### Sing‐Cell RNA‐Seq Analysis

The study of scRNA‐seq was approved by the Ethics Committee of Zhongshan Hospital, Fudan University (B2019‐178). The datasets can be found on NGDC Bioproject with accession number PRJCA007656. The R package Seurat (v4.3.0.1) was used to perform sing‐cell RNA‐seq analysis. Cells with <1000 transcripts and >6000 genes detected were filtered out as low‐quality cells. In addition, cells with over 10% of mitochondria derived transcripts were considered to be low‐quality cells and were also excluded. R package Harmony was used to integrate different samples. We next performed normalization, dimensionality reduction, cell clustering and cell type annotation. All cell types were identified by the special marker genes: nucleus pulposus cells (NPC) highly expressing COL2A1 and ACAN, progenitor NP cells expressing THY1 and CD70, M1 macrophages highly expressing CD86, M2 macrophages highly expressing CD163, T cells highly expressing CD3D and CD3E, Neutrophils highly expressing PTPRC and S100A8. R package ggplot2 (v3.5.1) was used to create the graph showing the cell proportion

### RNA Sequencing

Differentially expressed genes were detected through mRNA sequencing, including fold change analysis, KEGG pathway enrichment for upregulated/downregulated pathways, and Gene Ontology (GO) enrichment for cell functions. The first experiment included three groups: Control, IL‐1β, and IL‐1β+β_Man, each with five replicates. The second experiment involved NT siRNA and siPrkcg groups, with three replicates per group. mRNA sequencing was provided by Novogene (China). Briefly, according to the manufacturer's protocol, total RNA was extracted from NP cells using TRIzol reagent (Invitrogen, 15 596 026). The mRNA was then enriched, fragmented, and reverse‐transcribed into cDNA. The resulting cDNA libraries were sequenced, and the sequencing data was analyzed and visualized using an online data analysis platform (https://magic.novogene.com/customer/main#/loginNew).

### TBS

Gene‐specific DNA methylation was assessed by a next‐generation sequencing‐based BSP, according to our previously published method. Targeted Bisulfite Sequencing libraries were constructed using the Acegen Targeted Methyl Panel Kit (Acegen, Cat. No. AG0508) according to the manufacturer's protocol. In brief, BSP primers were designed using the Acegen self‐designed script. 500 ng of genomic DNA was converted using the ZYMO EZ DNA Methylation‐Gold Kit (Zymo Research, Irvine, CA, USA). The products were used as templates for PCR amplification with 10 cycles. For each sample, the products of multiple genes were end‐repaired, 3′‐dA‐tailed and ligated to 5‐methylcytosine‐modified adapters. The constructed libraries were then analyzed by Agilent 2100 Bioanalyzer and finally sequenced on Illumina platforms using a 150 × 2 paired‐end sequencing protocol.

Use BSMAP v2.7.3 to mapping target sequences. Then, BSMAP v2.7.3 is used to detect methylation levels at various sites on the amplicon. We counted methylated C sites under sequence environments (CG). To study between‐group differences, a t‐test was used to detect the site of each amplicon in each comparison group.

### TAS

Gene‐specific DNA methylation was analyzed using next‐generation sequencing‐based BSP, following our published method. Targeted Ace Sequencing libraries were created with the Acegen Targeted Hydroxymethyl Panel Kit (Acegen, Cat. No. AG1119). BSP primers were designed using Acegen's script, and DNA was converted with T4‐BGT for glucosylation of 5hmC and APOBEC deamination. These products served as templates for 10 cycles of PCR amplification. The products were end‐repaired, 3′‐dA‐tailed, and ligated to 5‐Hydroxymethylcytosine‐modified adapters. The libraries were analyzed with an Agilent 2100 Bioanalyzer and sequenced on Illumina platforms using 150 × 2 paired‐end sequencing. The analysis method is the same as for TBS.

### Acquisition of Human NP

This study was approved by the Ethics Committee of Qilu Hospital, Shandong University (KYLL‐2022(ZM)‐1015). All patients were informed about the experimental plan and their rights and provided written informed consent for the collection of NP during routine lumbar disc surgery for patients with lumbar disc herniation. The severity of IDD was assessed using the Pfirrmann grading system.

### Construction of the In Vivo Animal Model of IDD

The animal experiments were approved by the Animal Ethics Committee of Qilu Hospital, Shandong University (Ethical Approval No. KYLL‐2022(ZM)‐1015), and the guidelines of the Animal Ethics Committee conducted all procedures. The experiment was conducted in a sterile environment using Sprague‐Dawley rats at 4 weeks of age. The rats were anesthetized with isoflurane inhalation. Under X‐ray guidance, a 26G needle was inserted into the center of the intervertebral disc (C3‐4). The needle was rotated 360° within the disc, fixed for 30 s, and then removed. The sham group only penetrated the skin, while the defect group received normal saline injections. β_Man treatment group: 1 week later, β_Man was injected intraperitoneally using a syringe (Low: 2.5 mg kg^−1^ d^−1^ High: 5 mg kg^−1^ d^−1^), once every 2 days, for 8 weeks.

Prkcg knockout: AAV NC or AAV shPrkcg (Adeno‐associated virus type 5 (AAV‐5), 1 × 10^10^ viral genomes, Haixing Biosciences, China) injection was performed only once after the puncture, directly at the site of the punctured IVD. All procedures were strictly conducted under sterile conditions, and the experiment was repeated five times.

TET2 overexpression: OE TET2 and EV were encapsulated in adenovirus and injected into the punctured disc twice, once in the first week and again in the fourth week after puncture. All procedures were performed under strict sterile conditions, and the experiment was repeated five times. β_Man was also treated by intraperitoneal injection with a syringe (5 mg kg^−1^ d^−1^), once every 2 days for 8 weeks.

### Imagine the Evaluation of Animal Experiments

MRI and micro‐CT examinations of the rat tail were performed during 4‐ and 8‐weeks post‐surgery. Micro‐CT imaging was conducted using a Bruker SkyScan 2211 system (Belgium). The rat tail was scanned using a 3.0 T MRI system (Intera Achieva, Philips, Netherlands). The disc height index (DHI) was calculated using Image J software with the following formula (Figure S18, Supporting Information)

Where the variables represent specific measurements obtained from the images, which are analyzed using Image J software. The IVD was assessed using the Pfirrmann grading system based on the disc structure and signal intensity. The specific grading criteria can be found in Supplemental Information.

### Histological Evaluation of Animal Experiments

The IVD was collected 4 and 8 weeks after treatment. The samples were fixed with 4% paraformaldehyde and decalcified slowly and steadily with EDTA (0.5 M, Servicebio, China). After decalcification, the samples were dehydrated and embedded in paraffin. The paraffin blocks were then sectioned into 4 µm thin slices in the coronal plane. The sections were stained with H&E, Safranin O/Fast Green stain, and Sirius Red stain, and examined by polarized microscope (VS200, Olympus, Japan). The degree of intervertebral disc degeneration and histological scoring are detailed in Table S2, Supporting Information.^[^ ^23^ ^]^

### IHC of the Tissue

After deparaffinizing the paraffin sections, antigen retrieval was performed by incubating in pH 9.0 EDTA for 2 min. The sections were then treated with 3% H2O2 at room temperature for 20 min to block endogenous peroxidase activity. After washing with TBS, the sections were blocked with 10% goat serum (Boster, China) for 1 h. Then, the sections were incubated overnight at 4 °C with the anti‐Collagen2α1(1:800, 28459‐1‐AP, Proteintech, China), anti‐MMP13 (1:100, 18165‐1‐AP, Proteintech, China), anti‐INOS (1:100, A14031, Abclonal, China), anti‐Prkcg (1:100, bs3626R, Bioss, China), anti‐TET2 (1:100, a5682, Abclonal, China). The HRP‐conjugated secondary antibody (RGAR011, Proteintech, China) was applied to the corresponding primary antibody. Afterward, we used diaminobenzidine (DAB, Solarbio, DA1015) for staining, followed by hematoxylin for nuclear counterstaining. Finally, decorate with xylene for 30 min. The slides were sealed with neutral resin and scanned using the VS200 (Olympus, Japan). The positive area was quantitatively analyzed using ImageJ.

### Statistical Analysis

In this study, statistical analyses were conducted using SPSS 19 (SPSS Science Inc., Chicago, Illinois) and Prism 6.0 (GraphPad Software, La Jolla, CA, USA). All in vitro experiments were conducted with biological replicates (n ≥ 3), and in vivo experiments were performed with five independent replicates (n = 5). Quantitative results are presented as mean ± S.E.M. Comparisons between control and treatment groups were performed using a two‐tailed Student's *t‐*test. For experiments with more than two groups, one‐way ANOVA was applied, followed by Tukey's post‐hoc test. In cases of non‐parametric data or lack of homogeneity of variance, the Kruskal‐Wallis H test was employed, with subsequent pairwise comparisons using the Nemenyi test. A *p‐*value of less than 0.05 was considered statistically significant.

### Ethical Statement

All animal experiments in this study were performed in accordance with institutional guidelines and approved by the Laboratory Animal Centre of Qilu Hospital of Shandong University (KYLL‐2022(ZM)−1051).

## Conflict of Interest

The authors declare no conflict of interest.

## Author Contributions

X.K., H.G., and Y.Z. contributed equally to the work. X.K., H.G., Y.Z., and L.C. designed the research. X.K., H.G., and Z.L. performed the research and analyzed the data. X.K. and H.G. prepared the manuscript. Q.M., Q.L., K.S., Y.L., H.Z., K.L., Z.L., T.L., and R.H. conducted sample selection and data curation. Z.W., F.W., and L.C. revised the manuscript. All authors read and approved of the final manuscript.

## Supporting information



Supporting Information

## Data Availability

The datasets generated and/or analyzed during the current study are available from the corresponding authors upon reasonable request.

## References

[1] GBD 2019 Diseases and Injuries Collaborators , Lancet 2020, 396, 1204. 33069326 10.1016/S0140-6736(20)30925-9 PMC7567026

[2] L. Chen , Q. Sun , R. Chou , D. B. Anderson , B. Shi , Y. Chen , X. Liu , S. Feng , H. Zhou , M. L. Ferreira , Int. J. Surg. 2024, 110, 1411. 38085809 10.1097/JS9.0000000000000951 PMC10942240

[3] a) K. Luoma , H. Riihimaki , R. Luukkonen , R. Raininko , E. Viikari‐Juntura , A. Lamminen , Spine (Phila Pa 1976) 2000, 25, 487; 10707396 10.1097/00007632-200002150-00016

[4] Z. Li , H. Yang , Y. Hai , Y. Cheng , Mediators Inflammation 2023, 2023, 6210885. 10.1155/2023/6210885 PMC10125773 37101594

[5] S. Roberts , H. Evans , J. Trivedi , J. Menage , J. Bone Jt. Surg., Am. Vol. 2006, 88, 10. 10.2106/JBJS.F.00019 16595436

[6] Y. Teng , Y. Huang , H. Yu , C. Wu , Q. Yan , Y. Wang , M. Yang , H. Xie , T. Wu , H. Yang , J. Zou , Acta Pharm. Sin. B 2023, 13, 2269. 37250166 10.1016/j.apsb.2023.02.018 PMC10213799

[7] P. Y. Huang , Y. H. Juan , T. W. Hung , Y. P. Tsai , Y. H. Ting , C. C. Lee , J. P. Tsai , Y. H. Hsieh , Cells 2024, 13, 1701. 39451219 10.3390/cells13201701 PMC11505648

[8] Y. Y. Chang , M. Wang , J. H. Yeh , S. C. Tsou , T. C. Chen , M. Y. Hsu , Y. J. Lee , I. Wang , H. W. Lin , Food Funct. 2023, 14, 10896. 37990840 10.1039/d3fo03568a

[9] K. Li , L. Wu , Y. Chen , Y. Li , Q. Wang , M. Li , K. Hao , W. Zhang , S. Jiang , Z. Wang , Drug Des. Dev. Ther. 2020, 14, 5315. 10.2147/DDDT.S278414 PMC7718963 33293793

[10] K. W. Lee , K. J. Ryu , M. Kim , S. Lim , J. Kim , J. Y. Kim , C. Hwangbo , J. Yoo , Y. Y. Cho , K. D. Kim , Proc. Natl. Acad. Sci. U S A 2024, 121, 2318039121. 10.1073/pnas.2318039121 PMC10998605 38536750

[11] Y. Yang , X. Luo , Y. Wang , A. Xu , L. Peng , X. Zhang , Z. Wang , Y. Ying , K. Li , Biomed. Pharmacother. 2024, 177, 117074. 38972149 10.1016/j.biopha.2024.117074

[12] C. S. Lin , C. L. Lin , T. H. Ying , H. L. Chiou , C. H. Hung , W. S. Liao , Y. H. Hsieh , S. H. Kao , J. Cell. Physiol. 2020, 235, 8446. 32324277 10.1002/jcp.29688

[13] C. Yang , N. Sun , Z. Liu , X. Li , Y. Xu , K. Zhang , Psychiatry Res. 2016, 237, 72. 26921055 10.1016/j.psychres.2016.01.076

[14] H. Takahashi , N. Adachi , T. Shirafuji , S. Danno , T. Ueyama , M. Vendruscolo , A. N. Shuvaev , T. Sugimoto , T. Seki , D. Hamada , K. Irie , H. Hirai , N. Sakai , N. Saito , Hum. Mol. Genet. 2015, 24, 525. 25217572 10.1093/hmg/ddu472

[15] A. Kose , N. Saito , H. Ito , U. Kikkawa , Y. Nishizuka , C. Tanaka , J. Neurosci. 1988, 8, 4262. 3183723 10.1523/JNEUROSCI.08-11-04262.1988 PMC6569481

[16] A. Nakazono , N. Adachi , H. Takahashi , T. Seki , D. Hamada , T. Ueyama , N. Sakai , N. Saito , J. Biol. Chem. 2018, 293, 14758. 30093405 10.1074/jbc.RA118.002913 PMC6153279

[17] Y. Gan , J. Long , Y. Zeng , Y. Zhang , Y. Tao , Mediators Inflammation 2022, 2022, 9923204. 10.1155/2022/9923204 PMC9584741 36274974

[18] M. Radulovic , H. Yoon , J. Wu , K. Mustafa , M. G. Fehlings , I. A. Scarisbrick , Neurobiol. Dis. 2015, 83, 75. 26316358 10.1016/j.nbd.2015.08.021 PMC4674329

[19] a) C. Alba‐Delgado , S. Mountadem , N. Mermet‐Joret , L. Monconduit , R. Dallel , A. Artola , M. Antri , J. Neurosci. 2018, 38, 10489; 30355630 10.1523/JNEUROSCI.1294-18.2018 PMC6596258

[20] a) Y. Gong , C. Wang , Y. Jiang , S. Zhang , S. Feng , Y. Fu , Y. Luo , Cells 2020, 9, 144; 31936169 10.3390/cells9010144 PMC7016760

[21] J. Mlost , M. Kostrzewa , N. Malek , K. Starowicz , Int. J. Mol. Sci. 2018, 19, 342. 29364174 10.3390/ijms19020342 PMC5855564

[22] a) Y. Liu , X. Sun , L. Wang , Y. Dou , Y. Tian , T. Yu , Y. Zhang , Q. Zhao , J. Lu , Y. Feng , J. Wang , X. Liu , Y. Shang , C. Li , Q. Yang , Adv. Mater. 2024, 36, 2408678; 10.1002/adma.202408678 39221659

[23] L. Chen , K. Peng , H. Huang , Z. Gong , J. Huang , A. M. Mohamed , Q. Chen , W. T. Sow , L. Guo , K. Y. H. Kwan , B. Li , M. A. Khan , P. Makvnadi , M. Jones , S. Shen , X. Wang , C. Ma , H. Li , A. Wu , Therapy 2024, 34, 2316545.

[24] a) C. Ligorio , M. O'Brien , N. W. Hodson , A. Mironov , M. Iliut , A. F. Miller , A. Vijayaraghavan , J. A. Hoyland , A. Saiani , Acta Biomater. 2021, 127, 116; 33831573 10.1016/j.actbio.2021.03.077

[25] C. Fan , W. Wang , Z. Yu , J. Wang , W. Xu , Z. Ji , W. He , D. Hua , W. Wang , L. Yao , Y. Deng , D. Geng , X. Wu , H. Mao , J. Nanobiotechnol. 2024, 22, 301. 10.1186/s12951-024-02556-8 PMC11140985 38816771

[26] K. Zhang , L. Du , Z. Li , Z. Huo , L. Shen , S. Gao , Y. Jia , M. Zhu , B. Xu , Biomater. Res. 2024, 28, 0047. 38952714 10.34133/bmr.0047 PMC11214826

[27] K. Geng , X. Ma , Z. Jiang , J. Gu , W. Huang , W. Wang , Y. Xu , Y. Xu , Cell Biol. Toxicol. 2023, 39, 1577. 35982296 10.1007/s10565-022-09748-8

[28] Z. Chen , J. Song , L. Xie , G. Xu , C. Zheng , X. Xia , F. Lu , X. Ma , F. Zou , J. Jiang , H. Wang , Cell Death Differ. 2023, 30, 1957. 37438603 10.1038/s41418-023-01190-5 PMC10406905

[29] K. Hashimoto , R. O. Oreffo , M. B. Gibson , M. B. Goldring , H. I. Roach , Arthritis Rheum. 2009, 60, 3303. 19877066 10.1002/art.24882 PMC2788707

[30] H. I. Roach , N. Yamada , K. S. Cheung , S. Tilley , N. M. Clarke , R. O. Oreffo , S. Kokubun , F. Bronner , Arthritis Rheum. 2005, 52, 3110. 16200590 10.1002/art.21300

[31] B. Cong , Q. Zhang , X. Cao , Protein Cell 2021, 12, 165. 33085059 10.1007/s13238-020-00796-6 PMC7895883

[32] a) W. Wang , N. Yang , L. Wang , Y. Zhu , X. Chu , W. Xu , Y. Li , Y. Xu , L. Gao , B. Zhang , G. Zhang , Q. Sun , W. Wang , Q. Wang , W. Zhang , D. Chen , Cell Rep. 2024, 43, 113873; 38427557 10.1016/j.celrep.2024.113873

[33] J. E. DeNizio , B. J. Dow , J. C. Serrano , U. Ghanty , A. C. Drohat , R. M. Kohli , J. Mol. Biol. 2021, 433, 166877. 33561435 10.1016/j.jmb.2021.166877 PMC8005466

[34] L. Hu , Z. Li , J. Cheng , Q. Rao , W. Gong , M. Liu , Y. G. Shi , J. Zhu , P. Wang , Y. Xu , Cell 2013, 155, 1545. 24315485 10.1016/j.cell.2013.11.020

[35] A. Yazici , T. Yerlikaya , J. Orthop. Surg. Res. 2022, 17, 541. 36514168 10.1186/s13018-022-03444-3 PMC9749279

[36] S. C. Shen , H. C. Chen , H. K. Tsou , R. H. Lin , Y. T. Shih , C. W. Huang , C. L. Tang , H. T. Chen , C. C. Chang , C. Y. Tzeng , Medicine (Baltimore) 2023, 102, 32832. 10.1097/MD.0000000000032832 PMC9901959 36749265

[37] S. Tian , X. Chen , W. Wu , H. Lin , X. Qing , S. Liu , B. Wang , Y. Xiao , Z. Shao , Y. Peng , Exp. Mol. Med. 2024, 56, 408. 38316963 10.1038/s12276-024-01168-4 PMC10907345

[38] X. Zhao , Z. Sun , B. Xu , W. Duan , L. Chang , K. Lai , Z. Ye , J. Nanobiotechnol. 2023, 21, 317. 10.1186/s12951-023-02075-y PMC10478255 37667246

[39] Z. Han , K. Benlagha , P. Lee , C. S. Park , A. Filatov , M. G. Byazrova , H. Miller , L. Yang , C. Liu , Front. Immunol. 2024, 15, 1459527. 39445011 10.3389/fimmu.2024.1459527 PMC11496051

[40] a) G. Pearson , F. Robinson , T. Beers Gibson , B. E. Xu , M. Karandikar , K. Berman , M. H. Cobb , Endocr. Rev. 2001, 22, 153; 11294822 10.1210/edrv.22.2.0428

[41] Z. L. Dong , X. Jiao , Z. G. Wang , K. Yuan , Y. Q. Yang , Y. Wang , Y. T. Li , T. C. Wang , T. Y. Kan , J. Wang , H. R. Tao , Mil. Med. Res. 2024, 11, 28. 38711073 10.1186/s40779-024-00529-4 PMC11071241

[42] J. Wang , Y. Zhang , J. Cao , Y. Wang , N. Anwar , Z. Zhang , D. Zhang , Y. Ma , Y. Xiao , L. Xiao , X. Wang , Autophagy 2023, 19, 2409. 36858962 10.1080/15548627.2023.2186112 PMC10392742

[43] E. Shimobayashi , J. P. Kapfhammer , Curr. Neuropharmacol. 2018, 16, 151. 28554312 10.2174/1570159X15666170529104000 PMC5883377

[44] Q. Wang , X. Zhang , X. He , S. Du , Z. Jiang , P. Liu , L. Qi , C. Liang , N. Gu , Y. Lu , Anesthesiology 2020, 132, 1212. 32101975 10.1097/ALN.0000000000003194

[45] S. Jarius , B. Wildemann , J. Neuroinflammation 2015, 12, 167. 26377184 10.1186/s12974-015-0357-x PMC4574118

[46] C. A. Pilo , A. C. Newton , Front. Cell Dev. Biol. 2022, 10, 929510. 35800893 10.3389/fcell.2022.929510 PMC9253466

[47] M. R. Kelher , N. J. McLaughlin , A. Banerjee , D. J. Elzi , F. Gamboni , S. Y. Khan , X. Meng , S. Mitra , C. C. Silliman , J Leukocyte Biol. 2017, 101, 261. 27531930 10.1189/jlb.3A0813-420RRR PMC5166440

[48] I. Dikic , Z. Elazar , Nat. Rev. Mol. Cell Biol. 2018, 19, 349. 29618831 10.1038/s41580-018-0003-4

[49] M. Picard , O. S. Shirihai , Cell Metab. 2022, 34, 1620. 36323233 10.1016/j.cmet.2022.10.008 PMC9692202

[50] W. Zhang , G. Li , R. Luo , J. Lei , Y. Song , B. Wang , L. Ma , Z. Liao , W. Ke , H. Liu , W. Hua , K. Zhao , X. Feng , X. Wu , Y. Zhang , K. Wang , C. Yang , Exp. Mol. Med. 2022, 54, 129. 35145201 10.1038/s12276-022-00729-9 PMC8894389

[51] J. N. S. Vargas , M. Hamasaki , T. Kawabata , R. J. Youle , T. Yoshimori , Nat. Rev. Mol. Cell Biol. 2023, 24, 167. 36302887 10.1038/s41580-022-00542-2

[52] E. S. Silagi , Z. R. Schoepflin , E. L. Seifert , C. Merceron , E. Schipani , I. M. Shapiro , M. V. Risbud , J. Bone Miner. Res. 2018, 33, 338. 28940640 10.1002/jbmr.3293 PMC5947995

[53] V. Madhu , M. Hernandez‐Meadows , P. K. Boneski , Y. Qiu , A. R. Guntur , I. J. Kurland , R. A. Barve , M. V. Risbud , Autophagy 2023, 19, 1821. 36628478 10.1080/15548627.2022.2162245 PMC10262801

[54] Y. Song , H. Liang , G. Li , L. Ma , D. Zhu , W. Zhang , B. Tong , S. Li , Y. Gao , X. Wu , Y. Zhang , X. Feng , K. Wang , C. Yang , Autophagy 2024, 20, 809. 37876250 10.1080/15548627.2023.2274205 PMC11062375

[55] X. Zeng , Y. D. Zhang , R. Y. Ma , Y. J. Chen , X. M. Xiang , D. Y. Hou , X. H. Li , H. Huang , T. Li , C. Y. Duan , Mil. Med. Res. 2022, 9, 25. 35624495 10.1186/s40779-022-00383-2 PMC9137164

[56] S. Geisler , K. M. Holmstrom , D. Skujat , F. C. Fiesel , O. C. Rothfuss , P. J. Kahle , W. Springer , Nat. Cell Biol. 2010, 12, 119. 20098416 10.1038/ncb2012

[57] W. Y. Huang , S. D. Hsu , H. Y. Huang , Y. M. Sun , C. H. Chou , S. L. Weng , H. D. Huang , Nucleic Acids Res. 2015, 43, D856. 25398901 10.1093/nar/gku1151 PMC4383953

[58] X. Wu , Y. Zhang , Nat. Rev. Genet. 2017, 18, 517. 28555658 10.1038/nrg.2017.33

[59] W. A. Pastor , L. Aravind , A. Rao , Nat. Rev. Mol. Cell Biol. 2013, 14, 341. 23698584 10.1038/nrm3589 PMC3804139

