# Supplementary material for: β‐Mangostin Attenuates TET2‐Mediated DNA Demethylation of Prkcg in the Prevention of Intervertebral Disc Degeneration
Source: Adv Sci (Weinh). 2025 Jun 25;12(32):e05077. doi: 10.1002/advs.202505077 (PMC12407310; doi:10.1002/advs.202505077)
Supplement: Supplementary file 1 — Supporting Information [file ADVS-12-e05077-s001.docx]

**Supplementary Information**


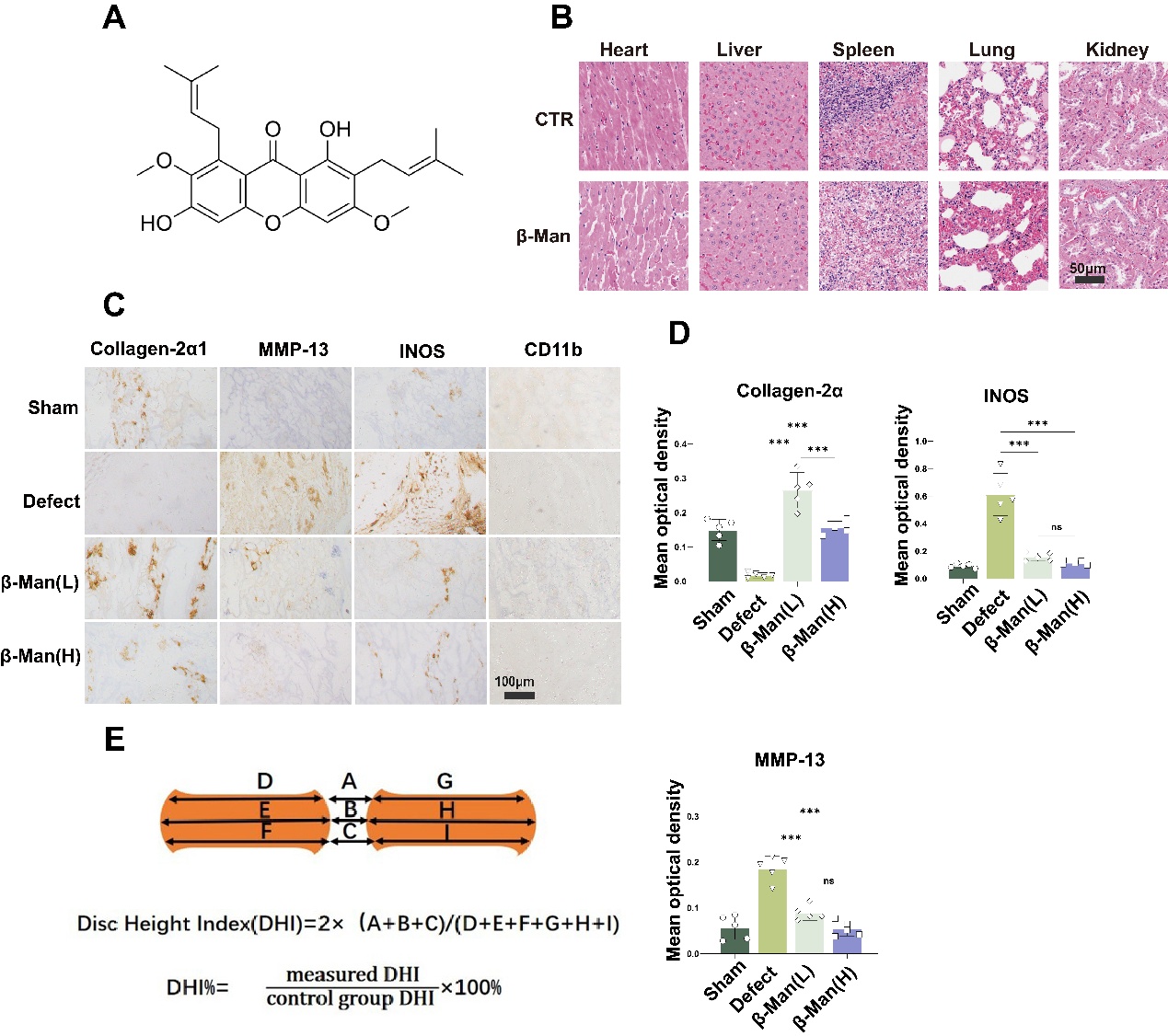


**Supplemental Figure 1**: β_Man can reduce ECM metabolism disorder in vivo. (A) β_Man chemical formula. (B) IHC staining of heart, liver, spleen, lung, and kidney to detect drug toxicity evaluation in rats (n=5, scale bar:50μm). (C) IHC of Collagen-2α1, MMP-13, INOS, and CD11b in different groups of rats (n=5, scale bar:100μm). (D) Statistical analysis of IHC. Data is presented as mean ± SD. (E) Method of DHI calculation. Data is presented as mean ± SD. ∗P < 0.05, ∗∗P < 0.01, ∗∗∗P < 0.001. ns, no significance.


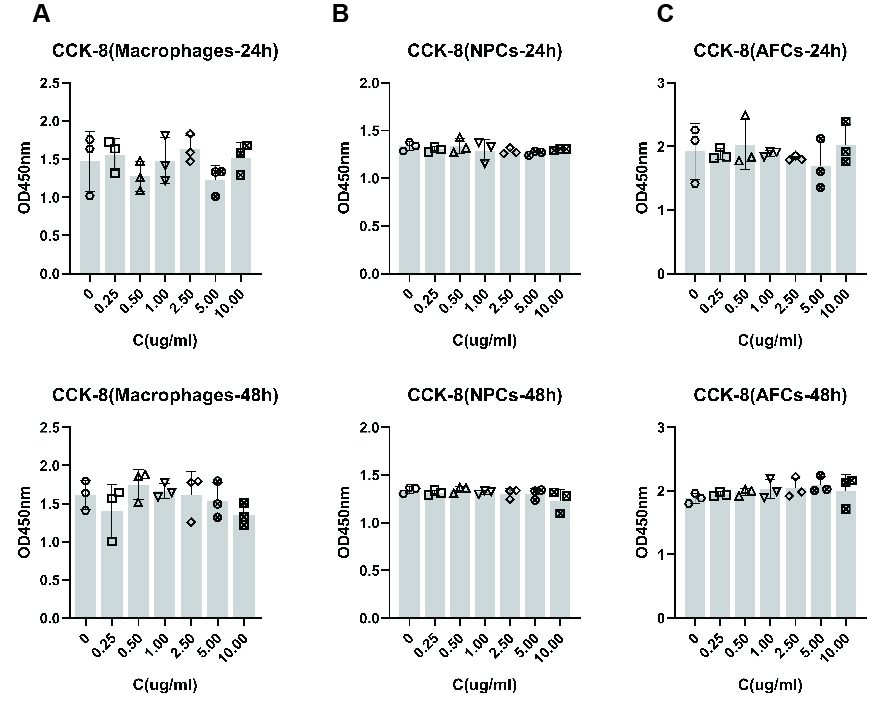


**Supplemental Figure 2**: CCK-8 results at 24h and 48h of macrophages, NPCs and AFCs. Data is presented as mean ± SD, n=3.


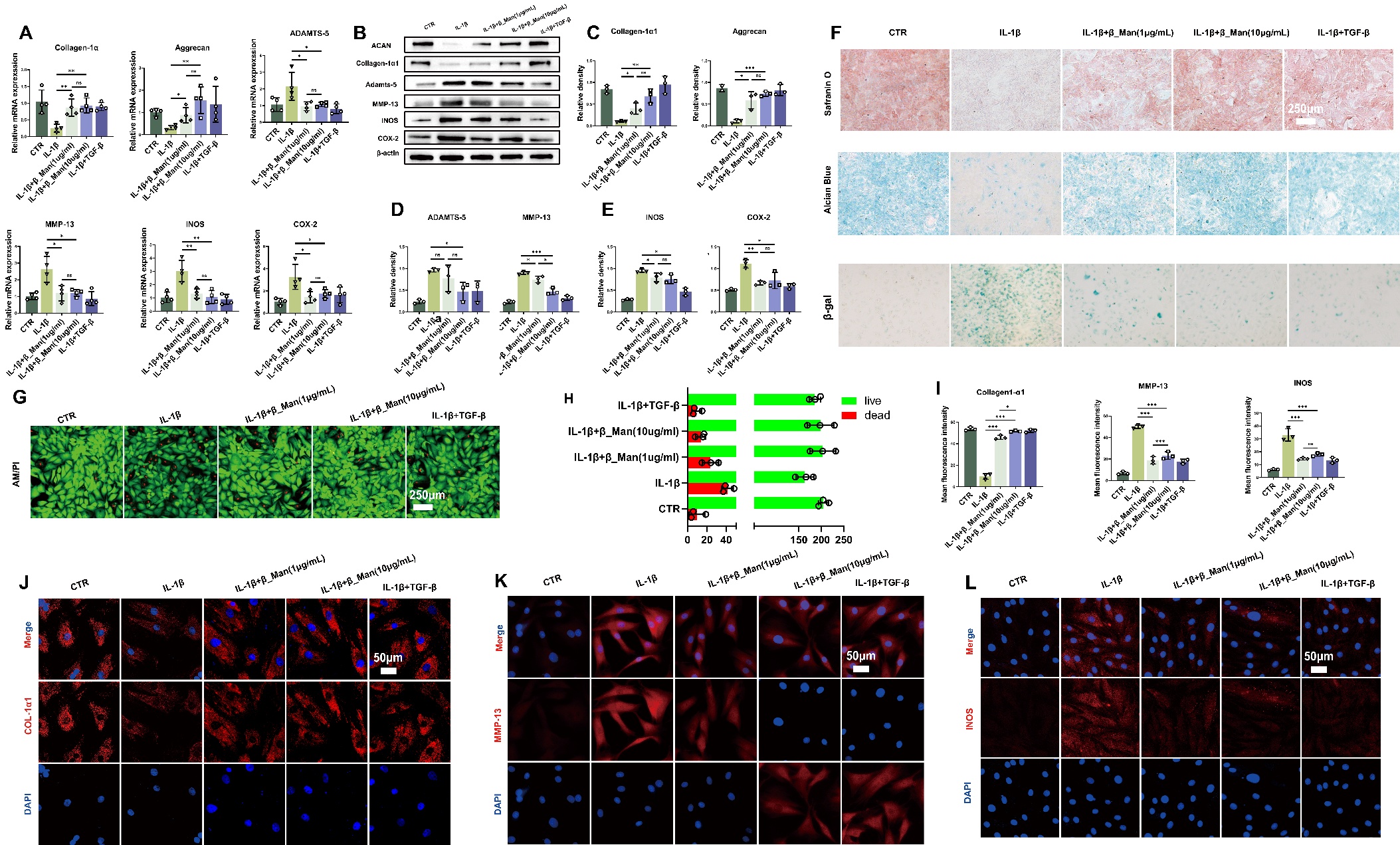


**Supplemental Figure 3**: β_Man improves AFCs ECM metabolism, alleviates cellular senescence, and reduces apoptosis. (A) PCR of ECM metabolism in AFCs treated with IL-1β, β_Man, or TGF-β (n=4). (B) Western blot shows the protein expression of the above genes (n=3). (C-E) Quantitative analysis of Western blot results. (F) Safranin O, Alcian Blue, and β-gal staining (n=3, scale bar:250μm). (G) Live/dead staining of AFCs (n=3, scale bar:250μm). (H) Quantitative analysis of live/dead staining results. (I) Quantitative analysis of IF. (J-L) IF staining of Collagen-2α1, MMP-13, INOS in NPCs (n=3, scale bar:50μm). Data is presented as mean ± SD. ∗P < 0.05, ∗∗P < 0.01, ∗∗∗P < 0.001. ns, no significance.


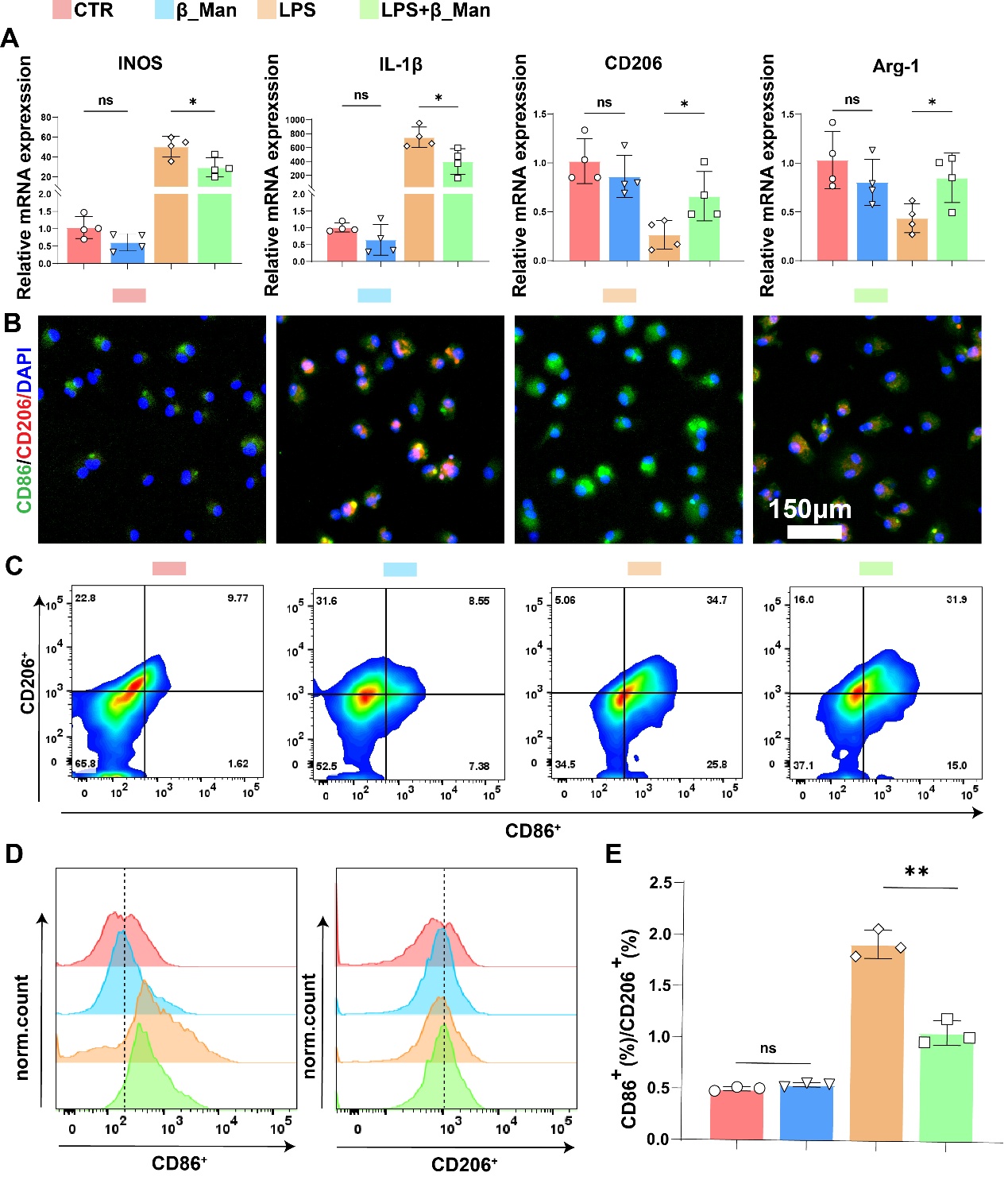
**Supplemental Figure 4**: β_Man alleviates LPS-induced M1 polarization in macrophages. (A) PCR analysis of INOS, IL-1β, Arg-1, and CD206 in macrophages treated with LPS or β_Man (n=3). (B) IF of CD206 and CD86 in macrophages (n=3, scale bar:150μm). (C) Flow cytometry of CD206^+^ and CD86^+^ macrophages (n=3). (D) Tree diagram analysis of the number of CD206^+^ and CD86^+^ macrophages. (E) Statistical analysis of the ratio of CD86^+^/CD206^+^. Data is presented as mean ± SD. ∗P < 0.05, ∗∗P < 0.01, ∗∗∗P < 0.001. ns, no significance.


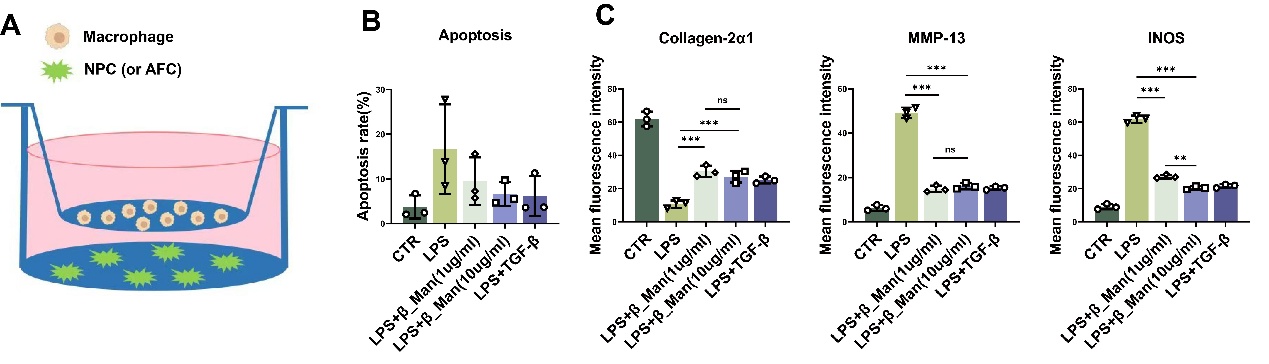


**Supplemental Figure 5**: (A) Diagram of co-culture of macrophages and NPCs (or AFCs). (B) Quantitative analysis of apoptosis results of NPCs (n=3). (C) Quantitative analysis of IF in co-culture system of macrophages and NPCs (n=3). Data is presented as mean ± SD. ∗P < 0.05, ∗∗P < 0.01, ∗∗∗P < 0.001. ns, no significance.


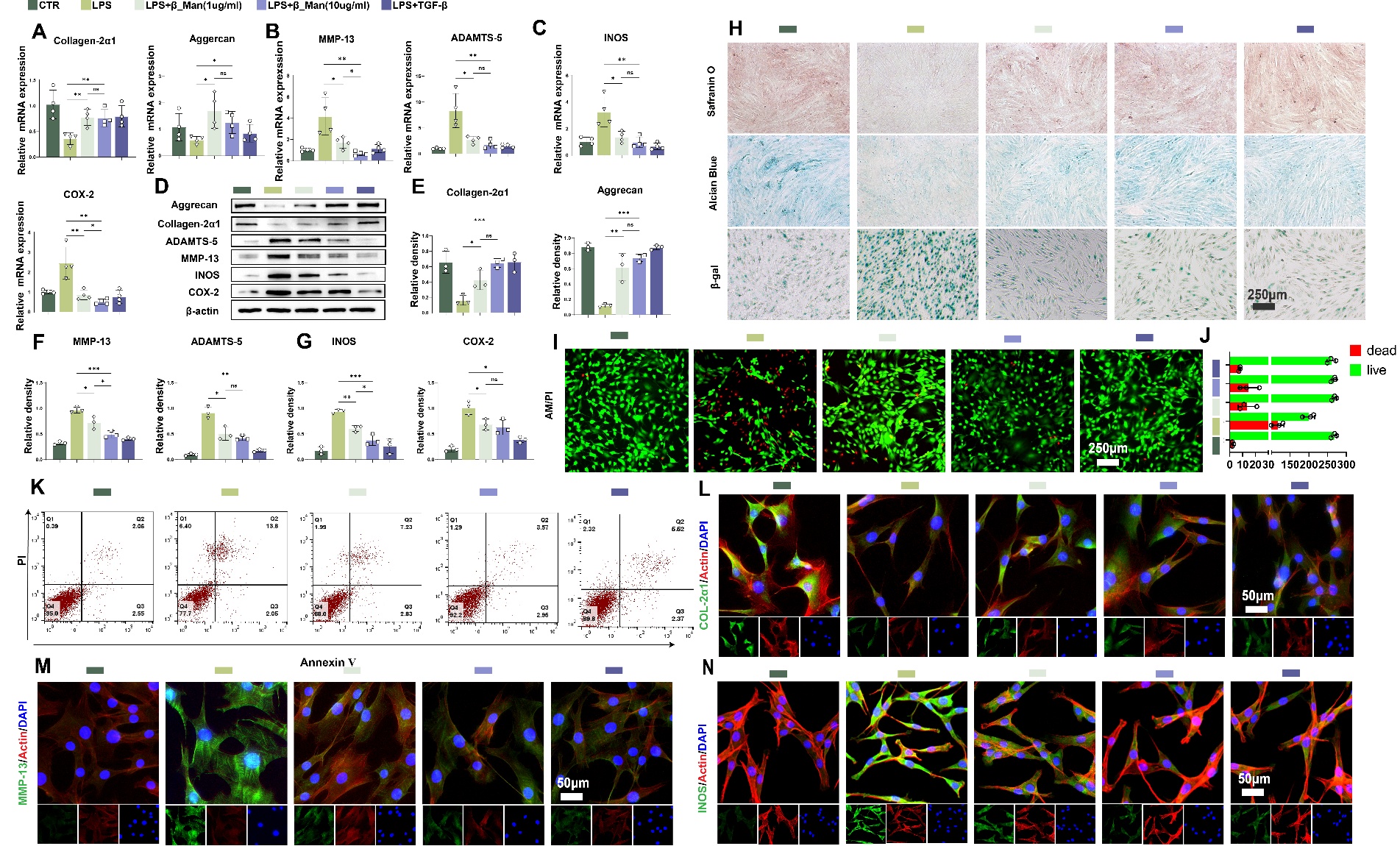


**Supplemental Figure 6**: **β_Man attenuates inflammatory damage by alleviating LPS-induced macrophage polarization in the macrophage-NPC co-culture system.** (A–C) PCR analysis of ECM metabolism (Collagen-2α1, Aggrecan), degradation genes (Adamts-5, MMP-13), and inflammation-related genes (INOS, COX-2) in NPCs treated with IL-1β, β_Man, or TGF-β in the system of NPCs and macrophage co-culture (n=4). (D) Western blot analysis shows the expression of the above genes at the protein level (n=3). (E-G) Quantitative analysis of Western blot results. (H) Safranin O, Alcian Blue, and β-gal staining of NPCs under different treatments (n=3, scale bar: 250μm). (I) Live/dead staining of NPCs (n=3, scale bar: 250μm). (J) Quantitative analysis of live/dead staining results. (K) Flow cytometry analysis of NPCs apoptosis (n=3). (L-N) IF staining of Collagen-2α1, MMP-13, INOS in NPCs under different treatments (n=3, scale bar: 50μm). Data is presented as mean ± SD. ∗P < 0.05, ∗∗P < 0.01, ∗∗∗P < 0.001. ns, no significance.


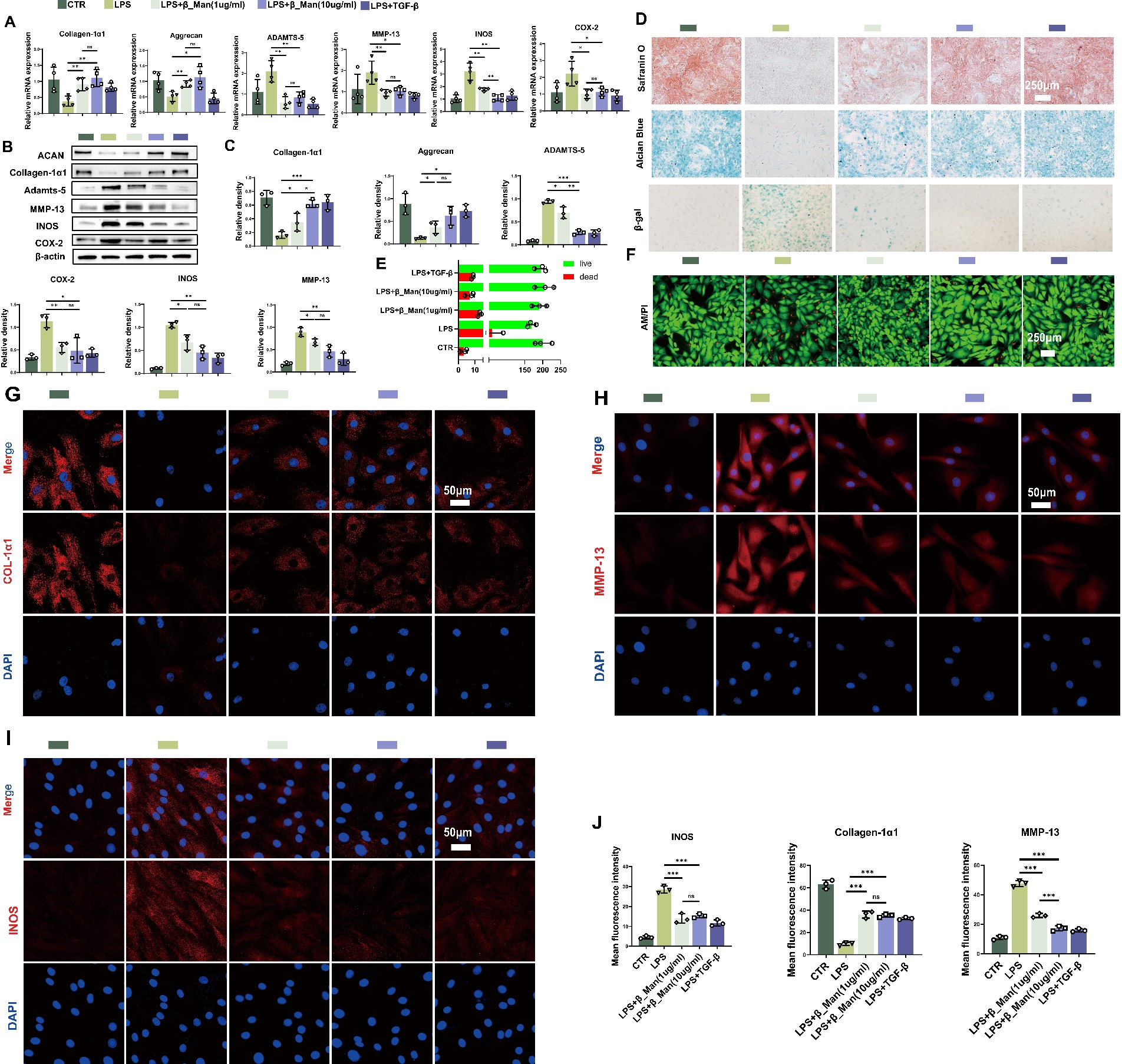


**Supplemental Figure** **7:** **β-Man protects AFCs by alleviating LPS-induced macrophage polarization in the macrophage-AFC co-culture system.** (A) PCR analysis of ECM metabolism in AFCs treated with IL-1β, β_Man, or TGF-β in the system of NPCs and macrophage co-culture (n=4). (B) Western blot analysis shows the expression of the above genes at the protein level (n=3). (C) Quantitative analysis of Western blot results. (D) Safranin O, Alcian Blue, and β-gal staining of NPCs under different treatments (n=3, scale bar:250μm). (E) Quantitative analysis of live/dead staining results. (F) Live/dead staining of AFCs (n=3, scale bar:250μm). (G-I) IF staining of Collagen-2α1, MMP-13, INOS in AFCs under different treatments (n=3, scale bar:50μm). (J) Quantitative analysis of IF. Data are presented as mean ± SD. ∗P < 0.05, ∗∗P < 0.01, ∗∗∗P < 0.001. ns, no significance.


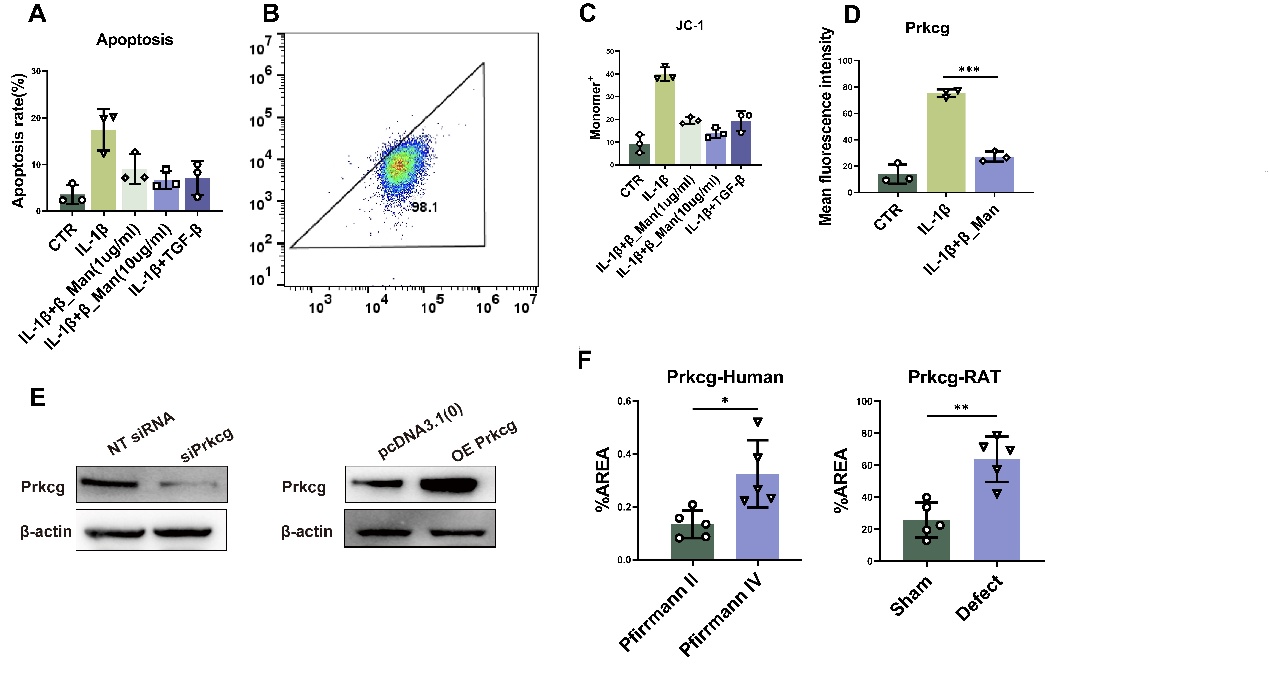


**Supplemental Figure 8**: (A) Statistical analysis of apoptosis of NPCs (n=3). (B) Flow cytometry of JC-1, the control group of CCP^+^ (n=3). (C) Statistical analysis of JC-1 (n=3). (D) Statistical analysis of IF of Prkcg in NPCs with IL-1β and β_Man (n=3). (E) OE/si Prkcg transfection efficiency validation. (F) Statistical analysis of Prkcg expression in human and rat NP by IHC (n=5). Data is presented as mean ± SD. n =3. ∗P < 0.05, ∗∗P < 0.01, ∗∗∗P < 0.001. ns, no significance.


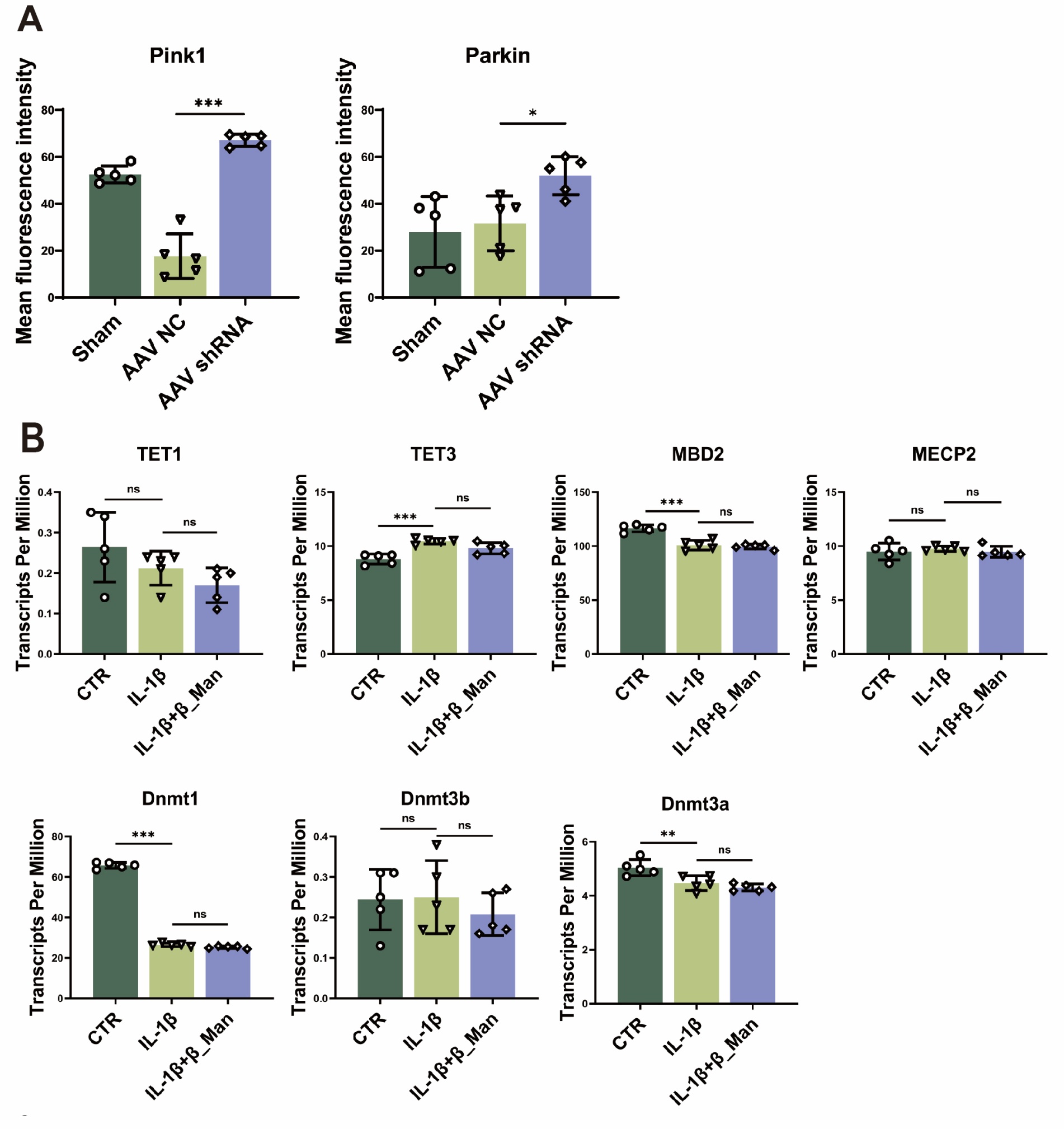


**Supplemental Figure 9**: (A) IF results of Pink1 and Parkin expression in IVD after Prkcg knockdown (n=5). (B) Methylation-related gene expression analysis (n=5). Data is presented as mean ± SD. ∗P < 0.05, ∗∗P < 0.01, ∗∗∗P < 0.001. ns, no significance.


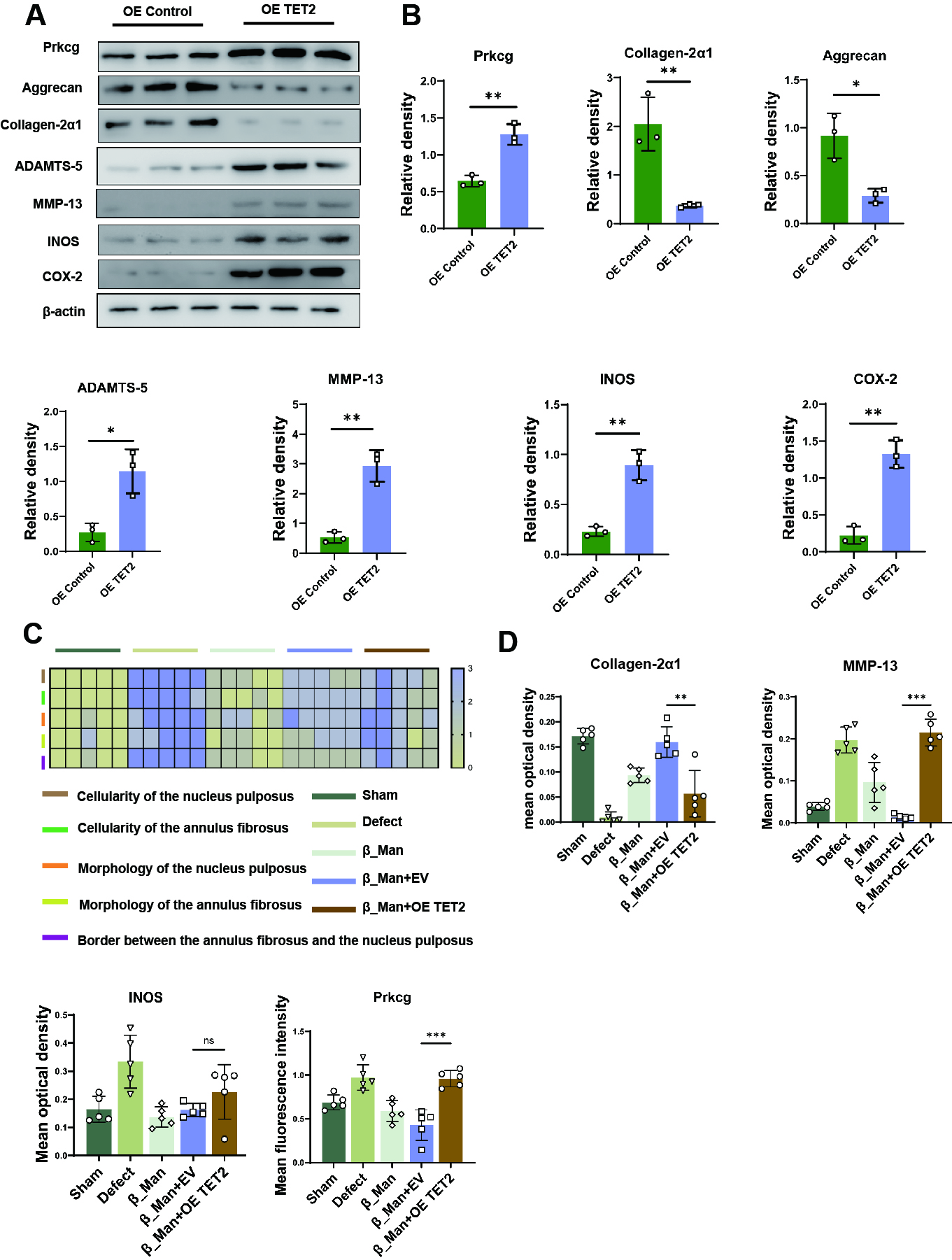


**Supplemental Figure 10**: (A) Western blot was used to detect the expression of different genes after TET2 overexpression(n=3). (B) Quantitative analysis of Western blot results. (C) Heatmap illustrating changes in histological scores across groups after TET2 overexpression (n=5). (D) IHC and IF results of Collagen-2α1, MMP-13, INOS, and Prkcg expression in IVD (n=5). Data is presented as mean ± SD. ∗P < 0.05, ∗∗P < 0.01, ∗∗∗P < 0.001. ns, no significance.

Table 1. Pfirrmann disc degeneration score

|  | I | II | III | IV | V |
| --- | --- | --- | --- | --- | --- |
| Structure | Homogeneous, bright white | Inhomogeneous with or without horizontal bands | Inhomogeneous, gray | Inhomogeneous, gray to black | Inhomogeneous, black |
| Distinction of NP and AF | Clear | Clear | Unclear | Lost | Lost |
| Signal  Intensity | Hyperintense, isointense to cerebrospinal fluid | Hyperintense, isointense to cerebrospinal fluid | Intermediate | Intermediate to hypointense | Hypointense |
| Height of IVD | Normal | Normal | Normal to slightly decreased | Normal to moderately decreased | Collapsed disc space |

| I. Nucleus pulposus morphology | II. Nucleus pulposus cellularity |
| --- | --- |
| Grade: | Grade: |
| 0: round shape, nucleus pulposus > 75% of disc area | 0: stellar-shaped cells |
| 1: round shape, nucleus pulposus = 50% -75% of disc area | 1: most stellar-shaped cells with some round cells |
| 2: nucleus pulposus = 25% -50% of disc area | 2: most round cells with some stellar- shaped cells |
| 3: nucleus pulposus < 25% of disc area | 3: round-shaped cells |
| III. Annulus fibrosus morphology | IV. Annulus fibrosus cellularity |
| Grade: | Grade: |
| 0: well-organized lamellae with no ruptures | 0: fibroblasts > 90% of the cells |
| 1: ruptured fibers < 25% of the annulus fibrosus | 1: fibroblasts = 75% - 90% of the cells |
| 2: ruptured fibers = 25% - 50% of the annulus fibrosus | 2: fibroblasts = 25% - 75% of the cells |
| 3: ruptured fibers > 50% of the annulus fibrosus | 3: fibroblasts < 25% of the cells |
| V. Border between the nucleus pulposus and annulus fibrosus | |
| Grade: |  |
| 0: Normal, without any interruption |  |
| 1: minimal interruption |  |
| 2: moderate interruption |  |
| 3: severe interruption |  |

Table 2. Histological grade scale of intervertebral disc

Table 3. PCR primer sequence(RAT）

| Gene | Primer sequence (F, forward; R, reverse; 5′-3′) |
| --- | --- |
| COL-2α1 | F: AGGAGACAGAGGAGAAGCT  R: CTTGAGGACCCTGGATTCC |
| Aggrecan | F: CACTTTACTCTTGGTCTTTGTG  R: AGTGAGTTGTCATGGTCTG |
| MMP-13 | F: ACCCAGCCCTATCCCTTGAT  R: TCTCGGGATGGATGCTCGTA |
| INOS | F: CACCTTGGAGTTCACCCAGT  R: ACCACTCGTACTTGGGATGC |
| COX-2 | F: AATCGCTGTACAAGCAGTGG  R: GCAGCCATTTCTTTCTCTCC |
| ADAMTS-5 | F: ACAACCAGCTAGGTGATGAC  R: AATGATGCCCACATAAATCCTC |
| CD206 | F: GAGGACTGCGTGGTGATGAA  R: CATGCCGTTTCCAGCCTTTC |
| Arg-1 | F: AAGACAGGGCTACTTTCAGGAC  R: ACCTTCCCGTTTCGTTCCAA |
| IL-1β | F: CCAGGATGAGGACCCAAGCA  R: TCC CGACCATTGCTGTTTCC |
| Prkcg | F: GGATAATGTGATGCTGGATGCT  R: CAATGATCTCAGGTGCTATGTAGT |
| TET2 | F: CCTATGATACAGATGACAGCACAA  R: TCCAGAAGAATGAGAACCAACAG |
| β-actin | F: CTCTGTGTGGATTGGTGGCT  R: CGCAGCTCAGTAACAGTCCG |

Table 4. PCR primer sequence(Human）

| Gene | Primer sequence (F, forward; R, reverse; 5′-3′) |
| --- | --- |
| Aggrecan | F: AAACCTGGCGTGAGAACTGT  R: CCACTGACACACCTCGGAAG |
| MMP-13 | F: ATTAAGGAGCATGGCGACTTCT  R: GCCCAGGAGGAAAAGCATGA |
| INOS | F: CGTGGAGACGGGAAAGAAGT  R: GACCCCAGGCAAGATTTGGA |
| GAPDH | F: GCACCGTCAAGGCTGAGAAC  R: TGGTGAAGACGCCAGTGGA |

Table 5. siRNA primer sequence

| Gene | Primer sequence (F, forward; R, reverse; 5′-3′) |
| --- | --- |
| si-Prkcg | F: GGGAGAGGUUGGAGAGACUtt  R: AGUCUCUCCAACCUCUCCCtt |
| si-TET2 | \| F: CCACACAGCCAAUGGGUUAtt \| \| --- \| \| R: UAACCCAUUGGCUGUGUGGtt \| |
